# A Digital Psychosocial Service (Sui App) for Arabic-Speaking Refugees in Switzerland: Development and Cultural-Contextual Adaptation Using a User-Centered and Participatory Approach

**DOI:** 10.2196/59905

**Published:** 2025-09-17

**Authors:** Rilana Tanja Stoeckli, Thomas Berger, Monia Aebersold, Viktoria Zoellner, Farhad Haji, Muriel Hunziker, Beatriz Jesus Ferreira, Michel Hosmann, Sebastian Burchert, Jessica Wabiszczewicz, Christine Knaevelsrud, Eva Heim

**Affiliations:** 1 Department of Clinical Psychology and Psychotherapy Institute of Psychology University of Bern Bern Switzerland; 2 Swiss Red Cross Bern Switzerland; 3 Department of Clinical Psychological Intervention Freie Universität Berlin Berlin Germany; 4 Department of Psychology University of Lausanne Lausanne Switzerland

**Keywords:** e–mental health, cultural adaptation, psychosocial support, digital MHPSS, refugees, participatory research, peer support, Arabic

## Abstract

**Background:**

Upon arriving in host countries, forcibly displaced people face psychological, cultural, as well as sociostructural challenges. Access to mental health and psychosocial support (MHPSS) remains limited, affecting both refugees and host country structures. Digital services offer promise in addressing these challenges, given their potential for scalability and accessibility. Despite the increasing use of digital MHPSS, cultural and contextual adaptation remains insufficiently documented, requiring systematic documentation.

**Objective:**

This study aims (1) to assess the psychosocial needs of newly arrived refugees in Switzerland and identify potential digital support solutions and (2) to participatorily develop and culturally adapt the Sui app to address those needs for Arabic-speaking refugees.

**Methods:**

We used a 2-phase adaptation process, structured using the RECAPT (Reporting Cultural Adaptation in Psychological Trials) framework to ensure systematic documentation. The preparation phase (2019-2021) included a desk review, needs assessment interviews, and conceptualization discussions. The development phase (2021-2022) included iterative development loops with a user advisory board (UAB) and a beta test, followed by final adjustments.

**Results:**

The desk review provided 5 key insights guiding development: mental health stigma, health literacy, sociostructural aspects, adherence to digital interventions, and task-shifting approaches. Findings from the needs assessment with 22 asylum care interviewees, 2 intercultural interpreters, and 4 target group interviewees confirmed the importance of integrating sociostructural and psychological factors. Through conceptualization discussions with a consultancy agency and 4 UAB (n=9) meetings, the scope of the Sui app was drafted: 9 relevant, everyday life topics (eg, asylum process, housing, and work), 5 psychological topics (eg, stress, sleep, and emotion regulation), and 1 peer-guided chat support feature. A multilevel translation process, involving 5 Arabic speakers from different countries, ensured linguistic accessibility. This allowed the UAB to focus on surface, content, and delivery during 19 development loops. The app’s content was delivered in various formats, including text, illustrations, video testimonies, and audio exercises. The beta test revealed high acceptability, with most users engaging with the app several times a week. However, technical challenges such as slow loading times hindered the full exploration of the features. Participants valued the peer support function, highlighting the importance of faster response times and personalized messages. Based on the beta test, technical and content refinements were made to prepare the app for a future quantitative evaluation.

**Conclusions:**

Through engagement with stakeholders and adherence to the RECAPT framework, we carefully considered the cultural and contextual circumstances of Arabic-speaking refugees newly arrived in Switzerland. The iterative participatory development process, spanning a preparation and development phase, ensured usability, accessibility, and relevance, while highlighting challenges in technical implementation and peer support. This study contributes to the existing knowledge of mental health and the needs of refugees and provides insights for future cultural adaptation of MHPSS interventions.

## Introduction

### Psychosocial Impact of Forced Displacement

Forced displacement, which happens primarily in low- and middle-income countries (LMICs), poses urgent humanitarian challenges worldwide. The number of individuals fleeing armed conflict, persecution, or environmental threats has increased to more than 108 million worldwide [1]. This mass movement imposes substantial challenges on the sociostructural dynamics on the side of host countries [2]. On the side of the affected individuals, the impact is apparent on several levels of their well-being.

Refugees and other forcibly displaced people may encounter psychological difficulties, including posttraumatic stress disorder (PTSD), anxiety, depression, psychosomatic disorders [3-8], and low quality of life [9,10]. Premigration traumatic events that influence mental health [11] are further compounded by life-threatening events occurring on the migration route [12]. Finally, in countries of destination, sociostructural impediments and cultural differences may deteriorate the potentially vulnerable mental health state in which refugees find themselves [13-16]. In the literature, these postmigration living difficulties (PMLDs) were found to have an impact on mental health beyond the effect of premigration events [14,17-22] or at least increase mental health difficulties [23-25]. In the ADAPT (adaptation and development after persecution and trauma) model, Silove [16] postulates the 5 core pillars—“safety and security,” “bonds and networks,” “justice,” “roles and identities,” and “existential meaning”—as a holistic approach to explain the interplay of past and ongoing challenges affecting psychosocial well-being of displaced people. Restoring those core pillars interdependently is explained to be crucial to promoting psychosocial recovery.

### Care Gap

Switzerland currently counts over 220,000 people in the asylum sector, including refugees and asylum seekers, with more than 20,000 from Syria alone. Recent asylum claims continue to come from 2 Arabic-speaking countries, Syria and Algeria, both of which rank among the top 5 countries of origin [26]. Disconcertingly, the critical need for psychological and sociostructural support is largely unmet, both in LMICs and in higher-income countries [2,27,28], such as Switzerland [29,30]. Specialists in Switzerland estimate that around 50% of refugees experience trauma-related psychological issues, with a large proportion untreated [30]. The limited access to essential mental health and psychosocial support (MHPSS) services is often caused on 2 levels. On the structural level, there is, for example, in Switzerland, a lack of mental health specialists working in the field of refugee mental health [30] and an immense overload weighing on social workers [31]. The latter results in fewer individuals being able to transition away from social welfare [32]. In addition, there is a lack of interpreters’ cost coverage [33], trained interpreters, childcare, and transportation (eg, to therapy) possibilities [28]. On the individual’s level, fear of stigma, language barriers, financial worries, and lack of self-perception of the mental health state are mentioned as barriers to psychological care [34-36]. For these reasons, the broad population of refugees, all of whom are facing major life changes, does not receive adequate psychosocial support [29]. More low-threshold psychosocial interventions are required, targeting the ADAPT model’s core pillars, PMLDs, and symptoms of mental distress to meet refugees’ psychological needs while taking into account their current life circumstances [16,25,37]. Swiss specialists have specifically recommended improving access to low-threshold services for individuals still living in asylum facilities [30].

### Scalable Approaches

The World Health Organization has developed community-based and accessible interventions (PM+ and SH+) to reduce symptoms of depression, anxiety, and PTSD [38]; to alleviate PMLDs [39]; to prevent mental disorders [40]; and to improve mental well-being [41]. A transdiagnostic treatment approach to reach refugees at the symptom level (the Common Elements Treatment Approach) was proven to be effective in 2 LMICs [42].

In Switzerland, while several low-threshold short interventions for distressed refugees have been documented, only a few have been tested through randomized controlled trials (RCTs) [43]. However, to our knowledge, no service has been evaluated that addresses the lack of social assistance within the context of psychosocial support. Given these persistent challenges in providing psychological and sociostructural support for refugees, exploring innovative solutions is imperative.

Emerging digital interventions have gained current and prospective research priority in humanitarian MHPSS settings [44]. Digital interventions have been shown to improve mental health difficulties such as depression, anxiety, posttraumatic symptoms, functioning, well-being, and quality of life among minority communities with large effect sizes [45]. A meta-analysis focusing on LMICs reported moderate to large effects of digital interventions on depression and anxiety, and a small impact on quality of life [46]. Digital mental health interventions offer the opportunity to overcome barriers such as limited structural or geographical access to specialists, language barriers, and fear of stigma [2,47]. This is particularly relevant for asylum seekers who, for example, in Switzerland, often reside in centers with limited access to urban areas [48], but usually have access to a smartphone [49,50]. Moreover, studies on internet-based interventions treating various mental disorders report comparable effectiveness as face-to-face interventions [51,52], with a conceivable advantage in cost-effectiveness [53] and a large potential for scalability [54].

The Lancet Commission on Global Mental Health has encouraged task-shifting interventions, including the involvement of nonspecialists in service provision, and advocates adopting digital platforms to scale up support for broader populations of people affected by mental ill health [55]. Internet-based interventions tend to be more effective [56] and have greater adherence [57] when they include human guidance, as opposed to unguided interventions. Interestingly, the professional education of the guide does not seem to be a critical factor [58,59]. The World Health Organization’s culturally adapted digital intervention Step-by-Step for treating depression trained nonspecialists to guide participants with weekly contact and demonstrated moderate effects in an RCT with Syrian refugees in Lebanon [60,61]. Using the task-shifting approach presents the opportunity to train peers who speak the target group’s first language and share similar experiences [62].

### Cultural and Contextual Adaptation

Studies focusing on culturally adapting psychological interventions aim to increase the acceptability and improve health outcomes for underserved target groups [63,64]. Cultural adaptation studies have mostly used “top-down” approaches in which pre-existing psychological interventions were adapted [65]. In contrast, “bottom-up” processes incorporate cultural aspects from the outset when developing new interventions [65].

A challenge of cultural adaptation remains the definition of the term. Resnicow and colleagues [66] explain cultural adaptation in 2 dimensions. On the observable dimension, so-called surface adaptations are made in order to increase acceptance. This includes visuals, audio, language, and delivery formats. In the second dimension, the deep structure is adapted. This includes a deep understanding of the social, cultural, historical, environmental, and psychological constructs that influence the health behavior of the target group. In the case of refugees, these constructs can be strongly influenced by various psychosocial pillars [16]. The term “culture,” therefore, does not only comprise the individual’s upbringing background (such as language, ethnicity, traditions, and country of birth). Culture is also dynamically shaped by recent experiences (eg, traumatic events during migration and interaction with the host community) and the current surroundings (eg, living in asylum centers, access to health care, and availability of digital devices).

Thus, we highlight the sensitivity to context as part of the cultural adaptation. In a Swiss study, for example, Syrian refugees most frequently reported social, cultural, and structural difficulties [67]. These included long waiting times for asylum decisions, travel restrictions, and challenges with diploma recognition, all of which are shaped by Switzerland’s specific legal and asylum system. While other European countries also present postmigration challenges, each country has distinct characteristics in its asylum and integration systems [68], requiring a context-specific understanding and adaptation of support services.

The benefits of cultural adaptation in psychological interventions are discussed in the literature. Some research has found superior effects of culturally adapted interventions compared to nonadapted interventions [69], whereas the extent of adaptation may play a role [70]. Other research suggests that the effects of psychological interventions do not depend on cultural adaptation [46,71,72]. In any case, it remains unclear what cultural adaptations of interventions have precisely entailed.

Various classification systems for reporting cultural adaptation to compare interventions have been developed for research [69-71,73]. Considering the inconsistent and varied classification methods, the RECAPT (Reporting Cultural Adaptation in Psychological Trials) framework was developed by a large consortium of researchers [65]. A growing body of studies adhering to standardized reporting criteria could enhance the replicability, comparability, and transparency of adapted interventions in the field of cultural clinical psychology [74].

### Objectives

In this paper, we report on (1) the assessment of needs and potential digital solutions for the mental well-being of recently arrived refugees in Switzerland and (2) the participatory development and culture-sensitive adaptation of the Sui app. Due to resource constraints, the first language version was specifically developed in Arabic, given the significant presence of Arabic-speaking refugees and asylum seekers in Switzerland. “Sui” stands for self-help, support, and information (in German: *Selbsthilfe*, *Unterstützung*, and *Information*), and the app is provided by the Swiss Red Cross (SRC).

To address the challenges of PMLDs that appear directly after arriving in Switzerland, we focused on refugees who have arrived in Switzerland less than 5 years ago. Our approach draws on participatory methodologies, which have been proven to be effective in tailoring health interventions for migrants [75]. Participation intends to establish sustainable partnerships and trust between the research and the target community. Participatory research empowers the voices of potential end users throughout the development and design process [76]. In software development, user-centered approaches are used as a form of participatory design and are characterized by iterative loops collaborating with end users [61,77]. We used this approach to deeply empathize with the needs and preferences of end users and adapt the service accordingly [61].

We plan to adapt the Sui app later to accommodate additional languages spoken by refugee communities. To approach inclusivity across different refugee backgrounds, we emphasize the importance of including context in the development and lay focus on bottom-up processes.

## Methods

### Overview

The Sui app was developed based on a user-centered, participatory, and mixed methods approach to provide a scalable, low-threshold MHPSS service for asylum seekers and refugees who have recently arrived in Switzerland. In the following, the term “refugees” is used inclusively to refer to both asylum seekers and individuals granted refugee status unless otherwise specified.

**Figure 1:**
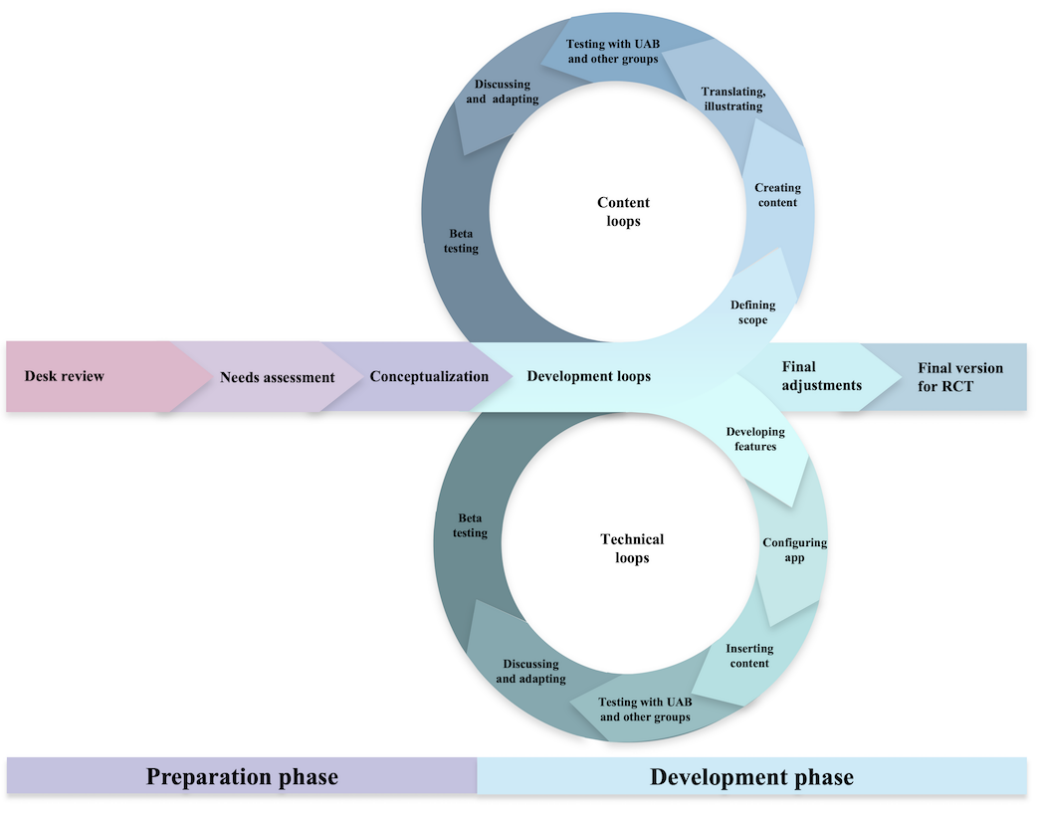
User-centered development process of the Sui app. RCT: randomized controlled trial; UAB: user advisory board.

Below, we describe the procedure of this paper in 2 major phases: *preparation* and *development* (see Figure 1). The documentation of cultural adaptation followed the RECAPT criteria by Heim et al [65]. See Multimedia Appendix 1 for the details on Chapter A: Set-Up; Chapter B: Formative Research; Chapter C: Intervention Adaptation; and Chapter D: Measuring Outcomes. In RECAPT, formative research is conducted in an iterative fashion before and during the process of adaptation. Formative research included a short desk review, as well as a broad stakeholder consultation at different stages. Interviews were analyzed using Mayring’s content analysis [78]. The documentation table, Chapter C (Intervention Adaptation) in Multimedia Appendix 1, was established from the beginning of the project to increase transparency and keep track of decisions made during the development process.

### Preparation Phase

#### Desk Review

First, a desk review of existing literature, based on the recommendation by Greene et al [79], was conducted. The aim was to rapidly gain an overview of the mental health and care situation of and for refugees in host countries and Switzerland in particular. The purpose of this desk review was to inform the development of the interview guideline for the needs assessment and the potential benefit of a digital mental health intervention. Specific questions that were addressed included the following: (1) What is the mental health situation of refugees? (2) What is the current mental health care situation? (3) What are the barriers for refugees to accessing mental health services? (4) What are the opportunities and risks of digital mental health interventions for the care of refugees?

To gather relevant literature, the ResearchGate database was searched for review articles and meta-analyses in German and English related to the mental health of refugees. The search included terms such as “mental health refugees Switzerland,” “psychosocial support asylum seekers,” “cultural adaptation digital interventions,” and “access to mental health care.”

The search was not exhaustive but was conducted with awareness of bias risks. The retrieved articles were screened for relevance based on their ability to address one or more of the key research questions. Findings from the selected literature were summarized under key thematic insights, grouping similar results together. The summarized insights were compiled into an unpublished internal report in December 2019.

A concise version of the desk review findings is provided in Multimedia Appendix 1 under Criterion 6, where additional literature was continuously documented throughout the process. In the Results section, we included only one key example source per main insight to maintain conciseness.

#### Needs Assessment

The needs assessment aimed to gain insights into the psychosocial circumstances of newly arrived refugees in Switzerland (within approximately 5 years of arrival) and explore the feasibility of a potential digital care tool. For this purpose, semistructured interviews were conducted by MA, RTS, and MH with psychotherapists, social workers, marketing and innovation professionals, as well as with residential care and health care professionals working in asylum contexts (asylum care interviewees [ACIs]), recruited within the SRC and associated organizations from the SRC network. Moreover, we included interviews with intercultural interpreters (ICIs) who live in Switzerland and also work for the SRC.

Additionally, we interviewed 4 people from the first language version target group (target group interviewees [TGIs]). The aim was to gain an initial insight into their perspective without striving for data saturation, as further engagement with the target group was already planned. The TGIs were informed about the opportunity to participate by their social counselor, who works for an SRC social service. A professional ICI translated the preparatory information as well as the interviews. For details on the interview guidelines, see Criterion 10 under Chapter D: Measuring Outcomes of Multimedia Appendix 1.

The recorded interviews were processed according to the summarizing content analysis described by Mayring [78], which is suitable for recognizing central statements. Additionally, Mayring [78] encourages researchers to consider the study’s macro context, including the cultural specialities of the research object. This aligns well with the culture-sensitive approach we followed with the RECAPT’s framework. In preparation of a similar cultural adaptation study for a digital intervention for Albanian immigrants in Switzerland [80], Shala et al [81] conducted a qualitative analysis according to Mayring [78] to evaluate the target group’s explanatory models. The needs assessment can be regarded as an explanatory model of psychosocial circumstances of refugees.

The first author (RTS), with an educational background in clinical psychology, analyzed the data. The analysis followed the subsequent five steps: (1) determination of units to be analyzed (recorded and transcribed interviews), (2) paraphrasing according to Z1-Z4 rules (reduction to essential and bundling of similar statements), (3) constructing the reduced statements into a category system, (4) re-evaluating the category system with the reduced material, and (5) finally interpreting the final categories.

To streamline and condense the content emerging from ACI interviews for this study, only categories with 5 or more codes from separate ACIs are reported with their corresponding frequencies. However, since the interviewees’ backgrounds were very heterogeneous, we considered all interview data for the conceptualization (for the entire collection of categories from ACI interviews, see Criterion 6 under Chapter B in Multimedia Appendix 1). The interviews with ICIs and TGIs were less structured, and the low number of interviews does not allow for the meaningful use of frequencies. However, the codes are reported in a summarized version. The needs assessments lasted from November 2019 until March 2020.

#### Conceptualization

In collaboration with a software consultancy agency, we deliberated on the desk review and needs assessment findings. With their help, the core team formulated the technical requirements and scoped out the project with agreed focus areas for the planned digital service. We used iterative design thinking strategies such as creating user personas, brainstorming, paper prototyping, and testing digital mock-ups [82]. Following this, we evaluated potential approaches to software development. The meetings were not transcribed verbatim. Instead, we drafted thought processes on paper and documented the key findings in Chapter C (Intervention Adaptation) of Multimedia Appendix 1.

To further refine the conceptualization and validate concept drafts, we established an Arabic-speaking user advisory board (UAB) representing the first language version target group. Members were recruited through outreach by the SRC, local aid organizations, and personal contacts using direct invitations and flyers. An ICI (coauthor FH), a key member of the core team, played a crucial role in forming and coordinating the UAB. He also acted as an intermediary between the UAB and the research team (see Criterion 2: Teams and Roles in Multimedia Appendix 1). The UAB discussions were held in Arabic and were also not transcribed verbatim. FH translated key discussion points following small group discussions, while another core team member documented them in protocols. These records were later added to the documentation table in Chapter C (Intervention Adaptation) of Multimedia Appendix 1.

The conceptualization findings were derived from prioritized topics from the needs assessment, their alignment with desk review findings, input from the UAB, and final consensus within the core team. Decisions were made while considering technical feasibility and scalability, in collaboration with the consultancy agency. The conceptualization was conducted between March 2020 and February 2021.

### Development Phase

#### Development Loops

In the development phase, the content and design were created according to the concept constructed during the conceptualization. The technical environment, as well as the content, including the design along with the text, was developed in loops involving various stakeholders: the core team, the asylum-information expert group, the translation group, the UAB, the Swiss Refugee Council, social services from the SRC, mental health experts, a design agency, a copywriter agency, a team from the Freie Universität Berlin, a software development agency and selectively other people. Previous cultural adaptation processes for digital mental health interventions followed a similar iterative development process, including translation, surface, content, and delivery or technical adaptations [61,80,83].

Based on the conceptualization, the chapters’ content was developed collaboratively. Each chapter was revised or partially written by respective experts (mental health experts, asylum-information experts, social services, and legal services). A professional interpreter translated the first version of each chapter, which was then revised by the translation group. This group was formed to cover understanding across various Arabic dialects. It consisted of 1 person each from Palestine, Tunisia, Egypt, Yemen, and Syria (2 women and 3 men). FH consolidated the feedback into a final translation.

The finalized translation was then revised in UAB meetings on surface, content, and technical revisions. Moreover, the UAB received homework (eg, reading texts or listening to audio exercises) to prepare for the meetings. Similar to the conceptualization meetings, development loop meetings for each chapter of the Sui app were conducted in Arabic, both in full-group and small-group discussions. Core statements of each small group were reported and translated at the end of the meetings by FH. These summaries were documented in protocols and later added to the Intervention Adaptation documentation table in Chapter C of Multimedia Appendix 1. The development loops took place between April 2021 and June 2022.

#### Finalization

In a beta test, participants completed an initial user experience (UX) test in a face-to-face meeting, tested the app for 4 weeks, and then took part in a face-to-face semistructured interview about the Sui app and optimal peer support at the end. The participants were recruited with the help of a local social counseling organization and the extended UAB and SRC networks. They had no prior involvement in developing the Sui app.

To evaluate how a contact person behind the app could be advantageous, a first concept for digital peer support was tested in the beta test with the aim of further developing it for the subsequent evaluation. This rough concept was based on the experiences of previous scalable interventions for refugees, such as Step-by-Step [84], Doing What Matters in Times of Stress [85], PM+ [86], and other internet-based treatments for depression and loneliness [87,88].

The SRC trained 2 Arabic-speaking people (beta test peers) with a migration background to provide a weekly message exchange with the app users. They were recruited following personal recommendations by the UAB. The training included getting familiar with the content of the Sui app and practicing writing support messages with standardized text templates, thereby using skills like active listening, paraphrasing, validating suffering, normalizing psychological symptoms of stress, and suggesting adequate subchapters of the app. They were also introduced to monitoring use behavior (completed subchapters) and activity (use time). Additionally, they had a supervision exchange with an Arabic-speaking psychotherapist. Findings from the beta test were used to understand the content and technical processes, develop a sensible structure for the motivational messages, understand the technological use in practice, evaluate the amount of support, and get an idea of what reactions to expect from participants.

The UX test, as well as the qualitative interviews, was conducted by 2 master’s students and coauthors (MH and BJF) and interpreted by FH from Arabic to German simultaneously. The beta test peers were interviewed in German, also with a semistructured interview at the end of the test period. For details on the UX test and interview guidelines, see Criterion 10 under Chapter D: Measuring Outcomes in Multimedia Appendix 1. The interviews were transcribed, and analyses were conducted following Mayring’s [78] content analysis with the same process as described for the needs assessment.

For the finalization, decisions and adjustments were applied based on the findings from the beta test. The peer concept was revised; technical and content app changes were made. The app was prepared to be used in a subsequent quantitative evaluation testing the effectiveness of Sui as a peer-guided or unguided service against a waitlist control group. The beta tests and the finalization lasted from July until October 2022.

### Ethical Considerations

Participants in the needs assessment (ACIs, ICIs, and TGIs) were invited to interviews verbally or in writing. They were given a short explanation of the planned project on digital mental health for trauma-affected refugees from all backgrounds living in Switzerland. Upon agreement, an interview was scheduled, and verbal consent was obtained for participation and audio recording, with recordings deleted poststudy. TGIs received a CHF 50 (approx US $55) supermarket voucher, while ACIs and ICIs were not incentivized. Ethical review was not sought, as participation was voluntary and noncritical.

For the conceptualization and development loops, UAB participants provided informed consent in Arabic, agreeing to the use of their summarized feedback in this paper. They received symbolic compensation of CHF 100 (approx US $110) supermarket vouchers. This research was approved by the local ethics committee of the Faculty of Human Sciences of the University of Bern in Switzerland (2021-09-00003).

Before the beta test, participants received study information in Arabic and sufficient time for review. They provided written consent for participation and audio recordings, which were deleted poststudy. The beta test was approved by the same local ethics committee (2022-06-00001) and conducted from June to August 2022.

All data remain fully anonymous, with no names, initials, or acronyms appearing in this paper or multimedia appendices.

## Results

### Preparation Phase

#### Desk Review

The desk review yielded 5 valuable key insights that contributed to shaping the further research questions. These were subsequently discussed in interviews with ACIs, ICIs and TGIs.

First, mental health issues often seem to carry a social stigma, leading many refugees to avoid seeking help due to fears of social consequences or beliefs that their condition is untreatable [89]. Addressing this stigma could potentially enhance the effectiveness of mental health interventions aimed at this population. Second, refugees exhibit diverse health literacies, understandings of illness, help-seeking behaviors, and treatment needs [90]. This suggests that mental health interventions may be most effective when tailored to align with the beliefs, values, and needs of the recipients. Third, interventions for refugees may need to extend beyond solely addressing mental health symptoms to consider the broader context of their living conditions [15]. It is conceivable that addressing only symptoms may not necessarily lead to an overall improvement in the quality of life for refugees. Fourth, the effectiveness of digital mental health interventions seems to hinge significantly on participants’ adherence and compliance, which are influenced by perceived benefits [91]. Thus, implementing various strategies to enhance adherence could have a positive impact on the effectiveness of interventions. Fifth, adopting a task-shifting approach shows promise for alleviating stress symptoms and improving refugees’ psychosocial well-being [55].

Moving forward, it was important to further explore the implications of these findings for implementing a digital mental health service aimed at improving the care situation of refugees in Switzerland, considering realistic opportunities and risks associated with such projects.

#### Needs Assessment

##### Findings of ACI Interviews

The results from the needs assessment with 22 ACI interviews are presented in 2 sections. First, the prevailing status characterizing the psychological state of refugees in Switzerland is described. Table 1 lists the psychosocial problems of refugees living in Switzerland, as identified by ACIs. Second, the potential of a digital tool to address the existing issues is elaborated. Table 2 illustrates the primary aspects highlighted by ACIs regarding the potential of a digital psychosocial support service. Subsequently, the summarized results from interviews with 2 ICIs (1 from Syria and 1 from Lebanon) and 4 TGIs (2 Syrians and 2 Syrian or Palestinians) are reported. Each interview lasted for approximately 60 minutes.

**Table 1:** Results on the current psychosocial problem situation in refugees residing in Switzerland: frequency of coding references (≥5) from the needs analysis found in semistructured interviews with asylum care interviewees working with refugees in psychological, medical, social, and residential care (N=22).

Categories		ACIs^a^, n (%)
**Main psychosocial problems**		
	Sleep disturbances	9 (41)
	Asylum situation (decision, rights, family reunification, and traveling)	9 (41)
	Physical pain or tension as an expression of psychological burden	8 (36)
	Occupational integration	8 (36)
	Social inclusion	6 (27)
	Housing	5 (23)
**Obstacles to healing**		
	Asylum situation	13 (59)
	Feelings of shame	7 (32)
	Fear of being categorized as “crazy”	6 (27)
**Resources**		
	Often strong resilience	11 (50)
	Daily structure and activities	8 (36)
	Social network	6 (27)
	Feeling of being needed (identity)	6 (27)

^a^ACIs: asylum care interviewees.

**Table 2:** Results on the potential of a digital psychosocial support service for refugees living in Switzerland: frequency of coding references (≥5) from the needs analysis found in semistructured interviews with asylum care interviewees working with refugees in psychological, medical, social, and residential care (N=22).

Categories					ACIs^a^, n (%)
**Concerns and obstacles toward digital tools**					
	No relationship and trust building			7 (32)	
	Lacking sensitivity of the digital tool toward emergencies and triggers			5 (23)	
	Lack of individuality			5 (23)	
**Potential use of a digital service**					
	Bridging tool (preparation, filling)			13 (59)	
	Support for ongoing therapy			9 (41)	
	Most people have a smartphone, and digitalization is future-oriented			8 (36)	
	Contact person behind the tool would be advantageous			8 (36)	
	No replacement for existing face-to-face services			7 (32)	
	Tool as a source for professionals and relatives of the affected			6 (27)	
	Emergency plan (advice)			5 (23)	
	Stabilization as primary goal			5 (23)	
**Digitizable content**					
	Psychoeducation			16 (73)	
	Psychoeducation on PTSD^b^ symptoms			8 (36)	
	**Information, explanations on sociostructural everyday aspects, and integration**			14 (64)	
		Housing	5 (23)		
		(Mental) health system	5 (23)		
		Occupational or educational integration	5 (23)		
	**Standardized exercises**				
		Body focused	14 (64)		
	Activation of existing resources			12 (55)	
	Connection to social life and activities			9 (41)	
	Asylum-related legal information			7 (32)	
**Important technical aspects to consider**					
	Balance between tailored and generic use			9 (41)	
	Anonymity: as priority and possibility to reach people			9 (41)	
**Advice on the delivery formats**					
	**Simple design and diverse formats**			15 (68)	
		Images and illustrations	7 (32)		
		Example stories	7 (32)		
		Short texts	5 (23)		
		Videos	5 (23)		
**Advice for psychological language within the tool**					
	Resource-oriented psychological language: normalizing, empowering, validating, taking seriously, hopeful, patient, humorous, listening, positive, and asking			10 (45)	
	Avoid stigmatized terms			7 (32)	
**Advice for better accessibility**					
	Participation of the target group in development and dissemination			9 (41)	
	Access through symptomatology			8 (36)	
	Access to information about everyday (asylum) life			6 (27)	

^a^ACIs: asylum care interviewees.

^b^PTSD: posttraumatic stress disorder.

##### Psychosocial Problems Reported by ACIs

In the interviews, the most prominent psychosocial challenges mentioned to be occurring in refugees were sleep disturbances, the overall asylum situation (including the asylum decision, process, and rights), physical pain or tension, occupational integration, social inclusion, and housing. Physical pain was frequently reported to serve as an expression of psychological burden in the target population.

Apart from being a psychosocial challenge, the asylum situation was mentioned as one main hindering factor for refugees to start a healing process or seek treatment since the focus is often on the structurally problematic situation. Other than that, feelings of shame and fear of being categorized or stigmatized as “crazy” were mentioned to be obstacles for people to express the need for help or seek treatment.

ACIs frequently mentioned that they perceive a resilience in many refugees that is difficult to describe but serves as a vital resource. In addition, a daily structure and activities, a social network and the feeling of being needed were mentioned as further concrete resources that contribute to improving the well-being of refugees.

##### Potential of a Digital Tool Evaluated by ACIs

Concerns of ACIs were that a digital tool could not build up a relationship or trust, which they perceive as essential in traditional psychotherapy. Another concern was that a tool could not detect emergencies or individual triggers and not react adequately. Similarly, they stressed that a digital tool cannot address individuality (eg, individual questions) properly.

However, a digital service was seen to be useful in approaching the present care gap. “The potential of the digital tool can lie in addressing the existing treatment gap, acting as a bridging, or filling additional service,” one ACI said. It was also envisioned as a tool supporting ongoing therapy, allowing clients to repeat information or exercises. In any case, a contact person behind the tool was considered advantageous for the users. According to the ACIs, a primary goal of the tool should be mental stabilization. Moreover, a digital tool was perceived as future-oriented, as most people own a smartphone. It was also recognized that the tool could benefit not only the target group but also professionals and relatives seeking information on psychosocial support for those affected. Nevertheless, it was emphasized that the digital tool cannot replace traditional face-to-face services.

Regarding the tool’s content, consistent suggestions were psychoeducation, particularly on symptoms related to PTSD. Typical psychotherapy exercises, especially body-focused exercises, were also deemed digitizable and effective. ACIs highlighted the importance of activating the individual’s existing resources (eg, asking about personal strengths). Along with psychological content, many ACIs suggested including sociostructural information typically provided by social services. Those include information about housing (eg, how to find an appropriate apartment), about the Swiss (mental) health system (eg, rights, existing services, and access), and occupational integration (eg, how to find a job or start an education). Additionally, a connection to “real” social life (eg, lists of communities, social clubs, and free activities) was considered indispensable. Besides providing information on everyday life, ACIs suggested integrating asylum-related legal information (eg, differences in residency status and family reunification).

Technical considerations of the tool involved striking a balance between generic use and individual tailoring. Anonymity was said to be paramount to ensure safety and to mitigate the fear of stigmatization toward psychological problems. Thanks to this anonymity, more people could be reached.

A consensus emerged on delivering content in a concise and straightforward manner, incorporating various formats, such as images, illustrations, example stories, short texts, and videos. It was advised that communication should adopt a resource-oriented approach: normalizing, empowering, validating, taking seriously, being hopeful, patient, humorous, listening, being positive, and asking. It was also stressed that the use of often stigmatized terms (eg, “patient” and “psychological disease”) should be avoided, even though they can vary across cultures.

Collaboration with the target group in both development and dissemination was strongly advocated. Furthermore, ACIs advised focusing on individual psychological symptoms as an entry point to the tool. Providing information on everyday life challenges that typically occur after migration can serve as another entry point to the tool.

##### Findings of ICI Interviews

The 2 ICIs reported similar psychological issues and PMLDs as the ACIs did. The reported problems included fear of being stigmatized as “crazy,” sleep disturbances, general mistrust, a lack of awareness regarding treatment options, worries about family left back home, residency status, and occupational integration challenges. One ICI explained that mental disorders are perceived as intrinsic to life and require time to pass. The other ICI stated that refugee traumatization should not be pathologized as a mental illness but rather viewed as adverse experiences that require strategies and possibly medication. Stigmatization, linguistic barriers, and difficulties in occupational and cultural integration were identified as obstacles to healing, while rapid networking within refugee communities and individual initiatives were recognized as resources. A digital support service should incorporate sociostructural assistance, such as links and addresses to social support resources, success stories, information on family reunification and other asylum-related topics, and general challenges encountered when arriving in Switzerland. Psychological content, including “dealing with mental disorders,” breathing exercises and their efficacy, and self-help techniques for crises, was also highlighted. The primary recommendation emphasized the significance of ensuring anonymity.

##### Findings of TGI Interviews

The main additional findings from the 4 interviews with TGIs were that they all use smartphones regularly, mainly to maintain contact with relatives, translate daily tasks, and access several consumer apps. Concerning psychological issues, all TGIs expressed that they will never be able to forget what they have survived and voiced concerns about the well-being of their family members left behind and have tried to reunite them in Switzerland. Some TGIs spoke about physical concerns that have occurred since their migration, alongside psychological challenges, such as stress, guilt, and shame. The encountered PMLDs among TGIs were as diverse as described by the ACIs, encompassing difficulties with language acquisition, residency status, occupational integration, waiting times, experienced discrimination, and navigating Swiss legal frameworks. Despite these challenges, all TGIs were able to identify positive aspects of their lives in Switzerland, citing factors like overall health, natural surroundings, happy people, tranquility, and being treated well. Reported resources included being grateful, having religious faith, fulfilling parental duties, ongoing efforts to reunite with family members, and receiving support from friends, family, or social counselors. Regarding the idea of a new support app, their imagination was limited to the knowledge of existing consumer apps and leisure activities. Consequently, they were rather unclear about the potential benefits of such a digital service.

#### Conceptualization

##### Overview

The UAB (n=9) participating in the conceptualization and subsequent development loops consisted of 8 participants and the key intermediary (FH) from Syria. The group was intentionally diverse in age (aged 19-55 years), ethnicity (4 Kurdish and 5 Arabic), educational background, and gender balance (4 women and 5 men). Four conceptualization meetings were conducted with the UAB, each lasting approximately 180 minutes.

The results from the conceptualization include decisions on technological aspects, the scope of the content, and its delivery formats. Additionally, a rough concept of peer-to-peer support to be used in the app is presented.

##### Technology

The digital service was decided to be delivered in the form of a cross-platform app that behaves like a native app, can be downloaded on iOS and Android devices, and is easily installable and updatable. At the time of making this decision, compared to progressive web apps, this format was seen as more accessible, robust, and better applicable on iOS devices for installation and receiving push notifications. Thanks to the compatible requirements, a mutually beneficial collaboration was agreed upon with the Department of Clinical Psychological Intervention at Freie Universität Berlin: Their “DIRECT” software can be used to create custom apps that can be adapted to the respective needs of the project and are specifically suitable for research in the field of mental health [92].

##### Scope of the Content

Regarding the content, the project team decided to combine sociostructural information with simple psychological well-being tools. The importance of focusing on sociostructural challenges in the asylum context was a key finding from the needs assessment (see above). Therefore, 9 chapters to provide sociostructural information and 5 psychological chapters were identified. The development of those chapters is described below. Following the recommendations of the ACIs, additional explanatory chapters were developed: national emergency contacts, introduction to the app, feedback on the app, and introduction of the storyline (for an overview, see Textbox 1). All content is made available directly after account setup since the target group and their needs are heterogeneous.

**Overview of the content of the Sui app. The Sui app is divided into 2 main sets of chapters, “Information” and “Well-Being,” and contains an additional “General” set.**

**Information chapters**

- Asylum Process
- Family Reunification
- Finances
- Health Promotion
- Health System
- Housing
- Residence Status
- Social Living
- Work and Education

**Well-being chapters**

- Stress
- Sleep
- Resources
- Chronic Pain
- Emotion Regulation
- Audio Exercises

**General chapters**

- Emergency Information
- Feedback
- Introduction
- Sui’s Neighborhood

##### Delivery Formats

Emerging from the needs assessment’s results, various delivery methods were decided to be used: simple texts, illustrations, stories, animations, explanation videos, video testimonials of refugees talking about personal experiences, audio exercises, list exercises, details of contact centers, and links to websites. We decided to create fictional characters to tell stories that our target group can identify with.

##### Peer Support

Due to significant concerns regarding security complexity, including the structured moderation of messages, we decided to abstain from implementing a feature that allows direct communication between users (eg, forum exchange). Nonetheless, the recommendation for a designated contact person within the app was strong. Given the demonstrated efficacy of guided formats in previous internet-based mental health intervention studies, we chose to integrate a guided version of the app. Furthermore, drawing insights from experience with prior peer-to-peer programs, we capitalized on the benefits of providing support through individuals sharing similar migration backgrounds with the target group (such as a shared language, cultural backgrounds, life experiences, and current challenges).

### Development Phase

#### Development Loops

##### Overview

The previously formed UAB (see the Conceptualization section) was involved in 6 meetings for design and surface discussions (prototyping and voting) and 13 meetings for content revisions (feedback and inputs on each chapter). Technical tests were conducted concurrently with content evaluations. The meetings lasted for an average of 210 minutes.

The reported results from the development loops only include a summary of the findings from the iterative development process. In the following, the results of this iterative adaptation process for design, language, and each chapter of the Sui app are described. Refer to Chapter C (Criteria 7, 8, and 9) in Multimedia Appendix 1 for a comprehensive description of the Sui app’s content, surface, and the associated adaptation and decision-making process. Over time, we divided the UAB into smaller groups so that discussions could be held in their native language (Kurdish or Arabic) and at different paces.

##### Illustrations

The bird “Sui” was introduced as the mascot of the app. Its name and color were accepted by all expert groups and the UAB, and it was preferred over a customizable avatar. The storyline around the app’s content introduces 12 fictional protagonists living together in an apartment block in Switzerland. They are from diverse sociodemographic backgrounds and countries, not only Arabic-speaking, to expand the app to other target groups at a later date. Their representation was evaluated by the UAB and by representatives of each of the characters’ ethnic groups. Each image was revised by the UAB, along with the corresponding content. Criticized features were adapted by the design agency and included, for example, lighter and more diverse skin colors, the use of a hijab, hairstyles, generally happier faces, change of clothes, or change of names.

##### Language

Originally produced in German, the written content was proofread by a Swiss copywriter agency to ensure simplicity and adherence to “plain language” principles. This involved minimizing complexity and shortening sentences, consistent with the recommendations from expert interviews (as reported above). We also found that the use of plain language facilitates subsequent translation and reading comprehension for people with low levels of education. Technical terms are explained in the glossary of the app and as a tooltip feature within the text. Arabic is an official language in more than 20 countries, including many countries that face political conflict and economic crises that often lead to the displacement of people. We, therefore, wanted to use an understandable version devoid of specific regional dialects. The translation group opted for a “Levantine Arabic+” that would be widely comprehensible across the Arabic-speaking population. We supplemented the initial Middle Eastern Levantine translation, done by a professional translator, with additional synonymous words suggested by the translation group. This approach aimed to achieve cross-national understanding. The UAB then made revisions and suggested minor changes. The intercultural translator integrated all feedback into a final version, focusing on an analogous translation and following the same principle as in the German version of offering the simplest possible language. This multilevel translation process was necessary to allow the UAB to understand every sentence and focus on the content rather than the language specifics. In addition, after UAB discussions, we agreed to add both a male and female Arabic version, as the second person pronoun (“you”) in Arabic distinguishes between two genders. The preferred form of address is set during account creation in the app.

##### Audio Exercises

Audio Exercises, featured in all psychological chapters and consolidated in one overview section, are drawn from several sources. However, most stem from the trauma-sensitive yoga founded by trauma therapist Härle [93-95]. The UAB deemed all exercises appropriate but generally preferred short durations. They particularly favored breathing exercises. Two voices, male and female, were chosen through a voting process involving the UAB and the translation group. Both female and male users hear both voices equally often. However, Arabic speakers will be addressed in either the female or male version of “you” based on preferences set during account creation.

##### Information Chapters

The 9 chapters on sociostructural information were developed based on existing first information material in collaboration with respective professionals, such as SRC social services, the Swiss Refugee Council, and an additional asylum expert group. The UAB revised each chapter. Because of the federal Swiss system, the depth of information was restricted to a national level since providing detailed information for each of the 26 cantons would have been impossible to implement simply. The chapters consist mainly of text-based step-by-step explanations but also include illustrations, video testimonies from refugees, and explanation videos.

The main results from the UAB meetings were as follows: housing covers everything important about the topic and includes valuable templates. However, they suggested incorporating more elements that convey hope for success into the content. For the “Work and Education” chapter, recommendations included adding more information on the education system, a job consultancy tool, a job platform, and instructions for CVs and application letters. Regarding the 3 asylum chapters, “Asylum Process,” “Residence Status,” and “Family Reunification,” they emphasized that highlighting accuracy due to prevalent misinformation is important. “Social Living” was deemed clear and important, with suggestions to list free activities, new events, and information about language courses. The “Health System” chapter should clarify where people can turn to for specific, how they can access care, and provide information on pregnancy and contraception equally for men and women. The chapter “Finances” was first considered too complex because several systems were not known in the UAB (eg, 3-pillar system, old-age and survivors' insurance). It was therefore expanded with information and was rated important, with suggestions for including money-saving ideas and differentiating between asylum social assistance and general social assistance. In the “Health Promotion” chapter, the UAB underscored the importance of avoiding any implications suggesting universal tobacco or alcohol consumption among refugees. Furthermore, they made recommendations on how healthy nutrition is possible despite a low budget.

##### Well-Being Chapters

The 5 chapters on psychological well-being were developed based on existing materials described below and adapted by the first author (RTS) according to the recommendations from the needs assessment. They were then reviewed by psychotherapists and researchers in the field of clinical psychology before the UAB gave feedback. The chapters consist of psychoeducation, written exercises, audio exercises, illustrations, and video testimonies from refugees.

The principal outcomes of the UAB revisions to each chapter were as follows: “Stress,” partly inspired by the DWM self-help guide [96,97], was considered important, mainly to understand the sources, to distract from, and maybe speak to someone about the experienced stress. “Sleep,” adapted from the content of a self-help book developed for traumatized refugees [98], includes sleep hygiene tips, body-focused exercises, and an introduction to sleep rituals. The UAB criticized using only smartphone-based exercises and recommended integrating exercises that can be carried out without a smartphone. The chapter “Resources” integrates planning activities, gratitude journaling, and strength activation, primarily inspired by the self-help book mentioned above [98]. It also features 2 subchapters on identifying values and staying present, derived from the DWM self-help guide [97]. Although the UAB deemed this chapter “not very important,” they expressed overall appreciation for its content and expanded the list of activities. “Emotion Regulation” aims to manage intense emotions using the “problem-solving therapy” by Nezu et al [99], which was tested in an e–mental health intervention for depression [100]. The shortened and adapted version of the Sui app focuses only on the first 2 noncognitive steps of “stopping” and “slowing down” before acting. It uses a traffic stoplight metaphor and presents 3 case examples of Sui’s neighborhood residents, whose relatable everyday life situations were appreciated by the UAB. For the “Chronic Pain” chapter, based on 2 self-help books [101,102], the UAB advised changing the psychoeducational texts into shorter and longer versions and rated the exercises as recommendable.

#### Finalization

##### Beta Test Setting

The Sui app was tested by 9 beta test participants, all of whom completed both the UX test and posttest qualitative interview. The group included 5 women and 4 men from Syria (mean age 28.44, SD 10.57 years), who had been living in Switzerland for 1 to 5 years. Of the 9 beta test participants, 5 were randomly assigned to receive weekly peer support during the testing period. The test peers were male and female (n=2, aged 29 and 33 years) and refugees in Switzerland coming from Syria and Egypt.

The results from the beta test, including the UX tests as well as the interviews, are summarized in the following. The UX tests averaged 45 minutes, whereas the interviews averaged approximately 60 minutes.

##### App Satisfaction

Most of the participants used the app several times a week and said that they were motivated to use the app. The functionality and structure of the app were unanimously rated as logical and simple. However, all participants complained about slow loading times during the interview and UX test. This led to reported problems accessing content or links within the app and app crashes. Most participants rated the illustrations as beautiful, appropriate, and compatible with the content. The illustrated protagonists were rated to be realistic, and participants could visually relate to them. Most participants rated the Arabic language as clear and understandable.

Half of the participants highlighted the chapters “Social Living,” “Housing,” and “Health System” to be helpful. Some participants rated “Sleep” to be good, particularly the exercises for falling asleep. However, some rated the included sleep hygiene tips to be superficial. Additionally, “Stress” and “Chronic Pain” were perceived as useful. Most participants appreciated the audio exercises, while a few had not used them. Some participants wished for more links, concrete realization examples, or more sociostructural chapters, for example, on drugs, forced marriage, or old-age and survivors’ insurance.

##### Satisfaction With Peer Support

With regard to peer support, beta test participants desired a quick response rate in the chat (6-72 hours). The messages should be formulated professionally but also in everyday language and adapted to the user. Support was also requested for technical issues. Some said that they would like to know some basic facts about the peer (eg, age, country of origin, and length of time in Switzerland) to be sure that they speak with a human and not with a computer. Having a peer companion was reported to enhance to motivation to use the app.

The test peers agreed that a 1-week response time was too long and should be shortened. They reported that, particularly for technical inquiries, they would suggest a response time of 48 hours. One of the test peers stated that most questions revolved around technical issues. Both stated that they would have preferred to write the messages more individually, and the use of text templates should be reduced, to provide the feeling of being a real person instead of automatic answers. The prior training was valued by both peers and rated to be important as a preparation.

Additionally, both agreed that continuous supervision and feedback on their work was important to seek help and reduce fears. Both highlighted that their messages motivated the users to use the app, and both tried to show their assigned participants that they are not alone.

##### Final Adjustments

**Figure 2:**
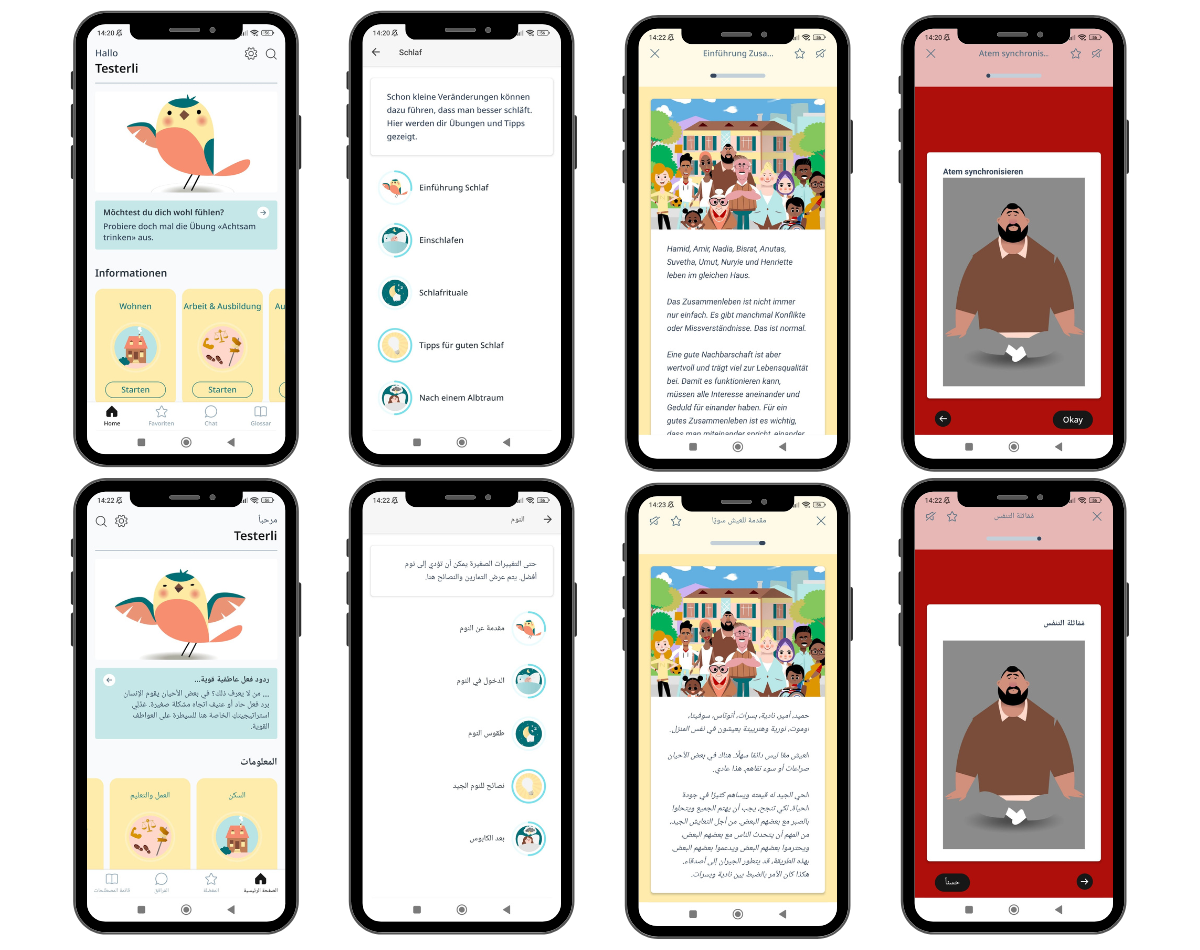
Screenshots of the final Sui app in German and Arabic.

Final adjustments on technical features and content were applied according to these beta test results before releasing a version for a subsequent evaluation. Loading times were improved, and the introductory chapter was expanded with explanations of features that were not self-explanatory (eg, explanation of the chat, the chapter sets “Information” and “Well-Being,” and the “Audio Exercises” chapter). For the peer concept, the peers’ general response time was reduced to 48 hours, and more intensive support for the peers regarding questions was introduced. The text templates were not reduced but rather complemented and rephrased to sound less “robotic,” as they were to remain standardized for comparability in the trial. The peers’ first messages will provide a more personal yet anonymous introduction, and peers will include their observations from the user behavior monitoring in their messages. Figure 2 displays screenshots of the final product.

## Discussion

### Principal Findings

In line with our objectives of (1) assessing the psychosocial needs of newly arrived refugees and identifying potential digital solutions and (2) developing and culturally adapting the Sui app in a participatory manner, we proceeded in two phases. During the preparation phase, a brief desk review and semistructured interviews revealed that newly arrived refugees in Switzerland face both psychological challenges (eg, sleep disturbances, stigma, and emotional distress) and sociostructural barriers (eg, navigating asylum procedure, securing housing, and occupational integration). These insights informed a first concept draft of the app. During the development phase, iterative consultations with Arabic-speaking advisors and expert stakeholders followed, resulting in the Sui app’s structure: 9 sociostructural chapters, 5 psychological chapters, and 4 general chapters. During these iterations, each chapter was refined (eg, simplified, reduced, and supplemented with examples), while content was translated into “Levantine Arabic+” with male or female pronoun options. Illustrations and stories, featuring the bird “Sui” as a mascot and 12 diverse characters, were added alongside audio exercises and video testimonials. Following a 4-week beta test involving 9 Arabic-speaking participants (5 of whom received peer support), the app was found to be highly acceptable: most participants used the app multiple times per week, particularly the chapters “Social Living,” “Housing,” and “Health System,” and they appreciated the peer-guided chat messages. However, slow loading times limited full engagement. We improved technical performance, added explanatory texts, and optimized peer support through faster responses and personalized messages, resulting in a more user-friendly Sui app ready for further evaluation.

Our finding that both psychological distress and sociostructural barriers coexist is consistent with prior research showing that PMLDs often exacerbate mental health symptoms beyond premigration trauma alone [13-16,23-25,89]. By embedding sociostructural alongside psychological chapters, we aligned the Sui app with the ADAPT model. This model posits that disrupted safety and security, bonds and networks, justice, roles and identities, and existential meaning interact to exacerbate distress, underscoring the need to address both psychological and sociostructural domains concurrently [16]. Furthermore, this consideration of context follows the recommendation that cultural adaptation should encompass not only background factors such as language or traumatic events, but also current concerns and stressors [61,103]. In contrast, existing smartphone-based MHPSS tools for refugees have focused predominantly on symptom reduction without integrating PMLD-related content [104].

Adhering to RECAPT guidelines [65], we combined surface adaptations with deep-structure adaptations, as has been done in other recent digital mental health interventions for immigrants [80,83,105]. The early integration of stakeholders across both preparation and development phases corresponds to a bottom-up and user-centered approach, which likely enhanced content and surface acceptability. This approach is supported by a review and guidelines on user engagement in digital mental health [45,106]. The well-perceived peer-guided chat aligns with broader global mental health strategies promoting task-shifting and human support in digital interventions [55,62], and with the successful implementation of other peer-provided mental health programs [39-41]. Participants’ preference for more personalized messages suggests that the standardized peer support should allow for flexibility to preserve authenticity while maintaining quality and consistency. Future research should examine the extent of personalization required to optimize user engagement and acceptability. Technical constraints (eg, slow loading times) were noted to be one of the main barriers to engagement in digital mental health studies [107], underscoring the value of beta testing predeployment. The intensive multistakeholder process required substantial time, financial resources, and coordination, echoing Shala et al [80]. However, the dual-phase design (preparation and development phase) created a modular core that should streamline future adaptation to other refugee language groups with similar needs in Switzerland, enhancing the potential scalability.

### Limitations

Various limitations of this study have to be considered. The focus on the participatory approach was placed on the development of the service, whereby the science-specific parts were not involved. These would have included the understanding of study information, the self-assessments, or the recruitment materials.

In the preparation phase, only 4 representatives of the target group were interviewed. As a result, we only had a limited subjective view of refugees at the beginning. It could have been advantageous to include a bigger population of TGIs in the preparation phase to receive a more subjective impression of the refugee experience in Switzerland. Additionally, the focus on digital support solutions in the needs assessment interviews may have influenced responses. Due to these reasons, its findings should not be generalized, but rather should be seen as initial insights.

Another limitation was that in initial UAB meetings, discussions were translated simultaneously, which led to a fragmented flow of conversation and, thereby, frustration among the participants. This process was changed as soon as concrete drafts of the content chapters were available. From then on, discussions were held in smaller groups, in Arabic or Kurdish, and each group presented the main discussion points and remarks in summary. The documentation of the discussions was, therefore, limited to these summaries in the end. A further limitation was that during the development loop discussions, some of the chapters were discussed multiple times with the UAB, whereas other chapters were only revised once due to time limitations.

Additionally, the beta test faced technical malfunctions, including long loading times and nonfunctional push notifications, such as those intended to indicate new messages from the test peer. Given a tight time plan, these difficulties could not be resolved in advance, significantly affecting the beta test results, as participants struggled to engage with the app’s content and primarily asked technical questions during their interactions with peers. This potentially prevented them from addressing other relevant issues. Furthermore, we did not assess quantitative metrics in the beta test, which limits our understanding of engagement patterns and barriers to adoption, which future studies should address by integrating a detailed tracking.

Another limitation was that the TGIs, the UAB, and the beta test included only participants from Syria, although we aimed to adapt the app for Arabic-speaking refugees from various countries. While different nationalities were included in the translation process to ensure broad linguistic accessibility, a more diverse participant group for user feedback would have strengthened the representativeness of adaptation needs.

While we used Mayring’s [78] qualitative content analysis to structure the data, thematic analysis might have provided a more flexible framework for capturing emerging themes and patterns across discussions. Future studies may consider applying thematic analysis to allow for a more interpretive approach when analyzing participatory data, also considering that there exists a larger amount of literature on thematic analysis with refugees [90].

These limitations could have led to a bias in several interpretations and decisions made during the development process.

### Conclusions and Implications

Forced displacement disrupts both psychological well-being and sociostructural stability. By addressing both psychological and sociostructural challenges, the Sui app represents a novel approach to digital psychosocial support for newly arrived refugees. Its task-shifting approach, that is, training peers to provide support via chat, can further improve engagement and effectiveness. Our iterative, participatory process ensured that content was not only linguistically accessible but also strongly responsive to refugees’ everyday realities and cultural understanding. Despite encountering challenges such as technical issues during the beta test, our findings affirm the potential of digital MHPSS services and the use of trained peers in bridging care gaps for marginalized populations. By thoroughly documenting our adaptation process, this study strengthens the evidence base for culturally adapting psychological interventions [65].

Based on our findings, several implications arise. First, digital MHPSS tools for refugees should include sociostructural information alongside psychological modules, as this dual approach concurrently better reflects refugees’ realities and enhances cultural relevance. Second, engaging target group representatives and other stakeholders from project inception through development ensures that content, language, and delivery format remain acceptable. Funders and implementers should allocate time and resources specifically for this multilevel adaptation. Third, training nonclinical peers to provide culturally appropriate support via chat can improve acceptability. Future research should examine which aspects of peer support most strongly drive positive outcomes. Finally, technical robustness must be prioritized through systematic beta testing and ongoing maintenance to optimize user engagement.

## Appendix

Multimedia Appendix 1 Completed template for documenting cultural adaptations of psychological interventions.

## Abbreviations

ACIs: asylum care interviewees
ADAPT: adaptation and development after persecution and trauma
DIRECT: Digital Research Creation Tool
ICIs: intercultural interpreters
LMICs: low- and middle-income countries
MHPSS: mental health and psychosocial support
PMLDs: postmigration living difficulties
PTSD: posttraumatic stress disorder
RCT: randomized controlled trial
RECAPT: Reporting Cultural Adaptation in Psychological Trials
SRC: Swiss Red Cross
TGIs: target group interviewees
UAB: user advisory board
UX: user experience

## Data Availability

The datasets generated and analyzed during this study are available from the corresponding author on reasonable request.
