# Supplementary material for: A Digital Psychosocial Service (Sui App) for Arabic-Speaking Refugees in Switzerland: Development and Cultural-Contextual Adaptation Using a User-Centered and Participatory Approach
Source: JMIR Form Res. 2025 Sep 17;9:e59905. doi: 10.2196/59905 (PMC12489417; doi:10.2196/59905)
Supplement: Multimedia Appendix 1 [file formative_v9i1e59905_app1.docx]

Supplementary materials

**Completed template for documenting cultural adaptations of psychological interventions**

According to Heim et al. (2021)

**Note: This is an abbreviated version. The complete RECAPT documentation for the Sui app can be requested:* [*rilana.stoeckli@unibe.ch*](mailto:rilana.stoeckli@unibe.ch)

# **Set-up**

- **Criterion 1: Definition of the target population**

| **Criterion 1: Definition of the target population** | |
| --- | --- |
| **Category** | **Results** |
| Target group | First language version target group for the subsequent evaluation:  The target population is defined as adult Arabic-speaking people living in Switzerland for five years or less.  The team of the Swiss Red Cross is aiming to adapt the intervention also for other target languages, such as French, Italian, English, Dari/Farsi, Turkish, Ukrainian, Russian Tigrinya and Tamil (exact choice of language is still to be decided). For this purpose, a balance between cultural adaptation to the Arabic-speaking population as well as the cultural adaptation to the asylum-related context had to be found. |

- **Criterion 2: Teams and roles**
- **Criterion 3: Documentation and monitoring system**
- **Criterion 4: Documentation of adaptations during the trial (“on-the-fly”)**

| **Criterion 2: Team and roles** | | | | | |
| --- | --- | --- | --- | --- | --- |
| **Team members** | **Decision maker group** | **Gender** | **Disciplinary background** | **Cultural characteristics** | **Role** |
| MA | Core team | f | Overall project leader, social worker in migration field | Swiss | Project leader, core team |
| VZ | Core team | f | Project leader “peers”, psychologist | Swiss / German | Project manager, core team |
| MH | Core team | m | Project leader “technical development”, psychologist, computer scientist | Swiss | Project manager, core team |
| RS | Core team | f | Psychologist, PhD student | Swiss | Psychological content, RCT conduction, core team |
| TB | Expert (e-)mental health | m | Psychotherapist, Prof. | Swiss | Research supervisor |
| EH | Expert (e-)mental health and cultural adaptation | f | Psychotherapist, Prof. | Swiss | Research supervisor |
| FH | Core team  Expert group “Arabic”  Key intermediary | m | Migration specialist  Intercultural interpreter/counsellor  Written translator | Syrian/Kurdish refugee, fluent in Arabic, Kurdish | Intercultural counsellor, core team |
| Intern during project development | Intern of the core team | f | Public Health student | Swiss | Intern, health system content |
| Experts on asylum-related topics | Expert group “asylum-information” | m/f | Social/migration/volunteer field in Switzerland | 1x Iranian  1x Swiss  1x Syrian | Revision of conceptualised content on the “information” part |
| Linguistic experts “Arabic” | Translation group | m/f | Language specialists | 1x Palestine  1x Tunisia  1x Egypt  1x Yemen | Translation control/adjustment group of Arabic translated scripts |
| User Advisory Board (UAB) | User Advisory Board | m/f | various | 3x Kurdish/Syrian  4x Syrian | Control group for concept, content, illustrations |
| Swiss Refugee Council (SFH) | Experts Asylum-related topics | f | Jurist | 1x Swiss | Providing information on legal issues |
| Swiss Red Cross (SRC) social services | Social-work-related topics |  | Social workers | Various, mostly Swiss | Providing information on cantonal differences |
| Free University of Berlin: SB, JW, CK | Technological collaboration, technical decisions | m/f | Psychologists, researchers | 2x German  1x German /Polish | Main software partner, expertise in tool development for research |
| **Support groups** | **No-decision-making authority** | **Gender** | **Disciplinary background** | **Cultural characteristics** | **Role** |
| Copywriter agency | - | f | Copywriter, journalist | No information available | Proof-reading of German scripts |
| Design agency | - | m/f | Illustrators, project leader | No information available | Story telling, illustration advice/realisation |
| Software consultancy agency | - | m/f | Innovation experts, UX/UI designers | Various, mostly Swiss | Support with conceptualisation, prototyping |
| BJF  MH | Supporting master’s students | f | BSc. In Psychology | 1x Swiss  1x Swiss / Portuguese | Pilot-phase leaders |
| Software developer agency | Supporting, advising technological decisions | m/f | Various fields of information technology | Various | Developing the software “DIRECT” on which the Sui app runs |
| Beta-test group | - | m/f | No information available | 5x Syrian  4x Syrian | Testing the Sui app over a time period of four weeks, with or without peer support |
| Test peers | - | m/f | Teacher  Digital artist | 1x Egyptian  1x Syrian | Supported half of the beta-test group during four weeks |
| **Formative qualitative research** | **No-decision-making authority** | **Gender** | **Disciplinary background** | **Cultural characteristics** | **Role** |
| Asylum care interviewees, ACI | Individual topic-specific experts | m/f | Psychologists, social workers, health care professionals in migration specific context | 22x mostly Swiss | Qualitative interviews before conceptualisation |
| Intercultural interpreters, ICI | Target group interviewees | m | Intercultural interpreters | 2x Kurdish Syrian  1x Lebanese | Qualitative interviews before conceptualisation |
| Target group interviewees, TGI | Target group interviewees | m/f | Various | 3x m Syrian  1x f Syrian | Qualitative interviews before conceptualisation |
| **Criterion 3: Documentation and monitoring system** | | | | | |
| The documentation of the main discussion points and decisions can be found under section C). | | | | | |
| **Criterion 4: Documentation of adaptations during the trial (“on-the-fly”)** | | | | | |
| This study documents the processes before the start of a randomised controlled trial. The authors agree to also adapt during the trial “on-the-fly” to improve the usability of the Sui app. | | | | | |

# **Formative Research**

- **Criterion 5: Formative research methods**

| **Criterion 5: Formative research methods** | |
| --- | --- |
| **Category** | **Results** |
| Desk review | A desk review of existing information on the target group’s well-being and psychopathology was conducted and summarised in an intern report by the Swiss Red Cross. The goal was to understand the key psychological issues faced by refugees. The desk review was expanded to include existing and evaluated digital mental health support interventions to gain insight into the existing literature on the impact and implementation of those interventions. |
| Qualitative methods | *Preparation:*  28 qualitative interviews were conducted with asylum care interviewees, target group representatives (Syrian refugees) and intercultural interpreters.  4 User Advisory Board meetings for brain storming, discussion, conceptualisation.  *Development and finalisation:*  6 User Advisory Board meetings for discussions around prototypes, topic choosing, exercise choosing, design agency choosing, speaker/voice choosing  Individual work of expert group “Arabic” for each session script  13 User Advisory Board meetings for revising developed content, illustrations  Beta test: test run with nine participants and two test peers  *On-the-fly:*  Reported bugs  Messages through peer chat  Calls from potential participants  Feedback function within the app |

- **Criterion 6: Target symptoms, syndromes, needs, and context**

| **Criterion 6: Target symptoms, syndromes, needs, and context** | | |
| --- | --- | --- |
| **Category** | **Results from the desk review and additions during the development** | **Source** |
| **Literature** | | |
| Context  *Post-displacement context in a high income host country* | **Selection of target group**  4 Arabic-speaking countries, Algeria, Syria, Morocco and Iraq amongst the top 10 nations where asylum requests in Switzerland stem from in 2022 (total of 3644 requests)  In 2024, out of around 220,000 people in the asylum sector, 20,000 are from Syria alone. Algeria and Syria remain among the top five nations of asylum requests.  The Arabic-speaking population of refugees therefore seems to remain one of the largest linguistic groups in Switzerland. | (Staatssekretariat für Migration, 2023, 2025) |
|  | **Problem situation**  High prevalence of mental disorders, especially PTSD and depression, as well as anxiety | (Blackmore et al., 2020; Hoell et al., 2021; Turrini et al., 2017) |
|  | Collective trauma: War in Syria caused a collective experience of violence, terror through torture, right violations and deprivation of basic needs | (Hassan et al., 2015) |
|  | People with refugee experience often refrain from seeking psychological help for various reasons (e.g., fear of stigmatisation, non-consciousness of mental state, different cultural concepts, etc.) |  |
|  | There are not enough services for the care of people with refugee experience in consideration of the high prevalence rate. Reflected in high waiting lists or rejection rates, mis-match of prevalence rate and therapy places, difficulties in payment of translators, etc. |  |
|  | **Pre-migratory stressors**  Pre-migration traumatic exposure is associated with poorer mental health.  These events include persecution, violence, torture | (Li & Anderson, 2016; Mesa-Vieira et al., 2022) |
|  | **Peri-migratory stressors**  Including traumatic experiences on the migration path, such as life threatening dangers (cold, heat, hunger, thirst, exploitation, humiliation, violence, etc.) |  |
|  | **Post-migratory stressors ave impact beyond pre-migratory stressors**  Postmigrational stressors positively associated with PTSD symptoms and  emotional distress | (Kaltenbach et al., 2018) |
|  | Post-migration living difficulties checklist | (Silove et al., 1997) |
|  | The post-migration stressors affect mental health (e.g., resettlements, language barriers, stigma, discrimination) | (Turrini et al., 2019) |
|  | Daily stressors and mental health are interconnected due to ongoing stress, loss of control, strongly affect the everyday life and are often very diverse (e.g., affecting housing, social life) | (K. E. Miller & Rasmussen, 2017) |
|  | Post-migration Living Difficulties predict changes over time in depression/anxiety | (Schick et al., 2018) |
|  | Daily stressors should be identified and addressed before specialized clinical care is being provided | (K. E. Miller & Rasmussen, 2010) |
|  | Emotional well-being is associated with language ability, employment status and accomodation satisfaction | (Campbell, 2018; Porter & Haslam, 2005) |
|  | In Switzerland, health-related quality of life has ben associated with the lack of social integration. Furthermore mental health difficulties predicted integration problems. | (Sijbrandij et al., 2017) |
|  | Long duration of the asylum process, family-related issues (separation, worries, loss, loneliness), social integration difficulties/low social participation, financial difficulties, unemployment, housing difficulties, language acquisition | (Gleeson et al., 2020) |
|  | Discrimination, unemployment, high PMLD: family-related issues, asylum procedure, socioeconomic living difficulties, socioreligious living conditions, loss of culture/support, adaptation difficulties, welfare/asylum | (Jannesari et al., 2020) |
|  | Interdependency of post-migration stressors/contextual factors and mental health and quality of life as well as help-seeking behaviour in high-income countries. | (Byrow et al., 2020; Hajak et al., 2021; Nickerson et al., 2011) |
|  | **Barriers to access health care**  Insufficient offer of professionals, resources | (Kiselev, 2020; Sijbrandij et al., 2017) |
|  | *Primary caregivers and mental health professionals in Switzerland say:*  Access barriers through financial struggles, fear of stigmatization, lack of information to available services. | (Bartolomei et al., 2016) |
|  | *On Syrian refugees living in Switzerland:*  Language barriers, gatekeeper problems, mismatch of problems and needs, lack of awareness, fear of stigma | (Kiselev, 2020) |
|  | Mistrust in public services | (Shannon et al., 2015) |
| Idioms of distress, specific target symptoms | **Conflict-related symptoms in Syrian refugees**  Armed conflict: Loss, grief, emotional, relational, material losses, ongoing concerns  Stress in displacement: isolation, feelings of estrangement, yearning for the lost homeland, loss of identity, discrimination against refugees, social tensions | (Hassan et al., 2015) |
|  | Emotional, cognitive, physical psychosomatic, social and behavioural consequences and mental disorders | (Renner et al., 2020) |
|  | **Psychological disorders in refugees**  Somatic distress in Syrian refugees is high | (Borho et al., 2021) |
|  | PTSD (9-36%), MDD (5%-44%), anxiety disorder (4-40%) | (Blackmore et al., 2020; Mesa-Vieira et al., 2022; Patanè et al., 2022; Silove et al., 2017; Steel et al., 2009; Thompson et al., 2018; Turrini et al., 2017) |
|  | Depression (up to 30%) | (Silove et al., 2017; Steel et al., 2009) |
|  | **Risk factors for adverse mental health outcomes of refugees**  Women, older age, low socioeconomic and educational levels, unemployment, low housing quality, dissatisfaction with accommodation, infrequent contact with relatives and friends, distrust of Western mental health practice, dissatisfaction with centrality of medicine in treatment, no language skills, social isolation, perceived discrimination and prejudice.  Lack of social networks, difficult job integration, complex asylum process, worries about family back home | (Farahani et al., 2021; A. Miller et al., 2018; K. E. Miller & Rasmussen, 2017) |
|  | **Main psychological symptoms in Syrian refugees**  *Emotional*: sadness, grief, fear, frustration, anxiety, anger, despair  *Cognitive*: loss of control, helplessness, worry, ruminations, boredom, hopelessness  *Physical*: fatigue, sleeping problems, loss of appetite, medically unexplained physical complaints  *Trauma-related*: nightmares, avoidance, hyperarousal  *Social*: withdrawal, aggression, interpersonal difficulties  🡪 conditions to promote strong resiliency is necessary to prevent functional impairment (current life circumstances)  *Substances:* Enhanced alcohol and drug abuse is suspected as a result of the crisis | (Hassan et al., 2015) |
|  | *Syrian refugees in Germany*  *Emotional:* hopelessness, longing for home/family, anhedonia, worthlessness, guilt, mistrust, helplessness, feeling foreign  *Cognitive:*  traumatic memories, nightmares, thoughts of homeland/family  *Physical*: sleeping problems, arousal symptoms, pain (headache, stomachaches) related to distress  *Social:* experiencing racism, loss of social network, loss of socioeconomic status, culture-related difficulties  *Behavioural:* withdrawal, aggression, avoidance, listlessness  *Mental disorders: depression, symptoms of PTSD* | (Renner et al., 2020) |
|  | **Syrian refugees on PTSD and depression in Norway**  PTSD as a word was not mentioned, symptoms were mentioned, such as:  Nightmares, difficult memories, thoughts of homeland and family  Depression was described with symptoms that were known but not mentioned with personal identification. Younger people mentioned the word “depression”, while older people rather spoke about “reaction to social problems”  Main stressors mentioned were: loss of social network, separation from family | (Aarethun et al., 2021) |
|  | **Most frequently mentioned psycho(somatic) problems**  *Physical:* Sleep disturbances (falling asleep, sleeping through, nightmares, getting up), physical pain often as expression of psychological burden (tensions, backaches, stomach-aches, headaches, dizziness)  *Social: S*ocial inclusion difficulties, feelings of loneliness, fear of something bad, never being able to forget what happened, worries about relatives left behind  These burdens were often summarized as “stress in general”. | Expert and target group interviews |
|  | Somatic distress is connected to psychopathology, traumatization and negative life events, and might be an expression of distress in general | (Rohlof et al., 2014) |
|  | **Post-migratory symptoms and difficulties**  *Syrian refugees in Switzerland*  *Psychological*: Symptoms of mental disorders, feelings of uncertainty, frustration, feelings of injustice, inferiority, isolation, distrust in system, feelings of not being understood, emotional impact of the war, marginalisation  *Structural*: residence permit, problems with government and authorities, problems with integration, cultural differences, language problems, problems related to education, employment, housing, providers | (Kiselev et al., 2020) |
|  | *Syrian refugees:*  *Structural* problems are most often mentioned: employment, government regulations, housing, finances, education, language  *Psychological* problems are mentioned secondly | (Drescher et al., 2021) |
|  | Often post-migration living difficulties (PMLD) are mentioned:  Loneliness, boredom or isolation, worries about family back home, being unable to return to home country in an emergency, difficulty learning German, separation from family, difficulties with employment, communication difficulties, being fearful of being sent back, difficulty obtaining financial assistance, difficulty obtaining appropriate accommodation, not enough money for food, rent or necessary clothes | (Aragona et al., 2012; Schick et al., 2016, 2018) |
|  | **Quality of life (QoL) of Arabic-speaking refugees**  *Syrian refugees in Germany:*  Low QoL in all domains (psychological, physical, social relationships, environment, WHOQOL-BREF) compared to a Western norm and Sub-Saharan population  Low QoL was associated with socioeconomic factors: housing, asylum duration, marital status | (Al Masri et al., 2021) |
|  | *Asylum seekers, refugees in Sweden:*  Low QoL in all domains (WHOQOL-BREF)  Social networks, social integration associated with higher QoL  Mental disorders associated with lower OoL | (Leiler et al., 2019) |
|  | *Asylum seekers, refugees in high-income countries:*  Post-migratory stressors are related to poorer quality of life:  Post-migration socio-economic status, weak social networks, poor social integration | (van der Boor et al., 2020) |
|  | *Newly- arrived female refugees in Australia:*  Overall good quality of life reported with social capital as main predictor and beyond trauma and PMLD | (Correa-Velez et al., 2010) |
|  | *Syrian refugees in Sweden:*  Financial and social strain are negatively correlated with quality of life | (Sengoelge et al., 2022) |
|  | Support on the level of needs, considering social life, support and psychosocial support.  This can prevent that many affected people will need specialized clinical services and identify needs earlier. | (Purgato et al., 2018) |
| **Note: This is an abbreviated version. The complete RECAPT documentation for the Sui app can be requested:* [*rilana.stoeckli@unibe.ch*](mailto:rilana.stoeckli@unibe.ch) | | |
| **Qualitative needs assessment: Collection of all categories emerging from a summarizing qualitative content analysis**  Asylum expert interviews (N = 22) | | |
|  | **Main psychosocial problems** | Semi-structured interviews with asylum care interviewees, consisting of psychotherapists, social works, marketing, and innovation professionals, as well as residential care and healthcare professionals working in asylum contexts |
| ACI = 9 | Sleep disturbances |  |
|  | Asylum situation (decision, rights, family reunification, traveling) |  |
| ACI = 8 | Physical pain/tension as an expression of psychological burden |  |
| ACI = 8 | Occupational integration |  |
| ACI = 6 | Social inclusion |  |
| ACI = 5 | Housing |  |
| ACI = 4 | Concentration difficulties |  |
| ACI = 4 | Feelings of insecurity related to asylum situation |  |
| ACI = 3 | General fear, panic |  |
| ACI = 3 | Trauma: triggers, flashbacks, disorientation |  |
| ACI = 2 | Strong sense of justice can be straining |  |
| ACI = 2 | Aggressive behavior |  |
| ACI = 1 | Irregular school attendance |  |
| ACI = 1 | Psychological pressure of suffering and not knowing where it comes from |  |
| ACI = 1 | Compulsive walking |  |
| ACI = 1 | Depression |  |
| ACI = 1 | Stress |  |
|  | **Obstacles to healing** |  |
| ACI = 13 | Asylum situation (decision, rights, family reunification, traveling) |  |
| ACI = 7 | Feelings of shame |  |
| ACI = 6 | Fear of being categorized as «crazy» |  |
| ACI = 4 | Guilt about being a survivor |  |
| ACI = 3 | Lacking language / Language barriers |  |
| ACI = 3 | Ongoing threat in home country, worries about family members |  |
| ACI = 3 | No peer exchange about mental health |  |
| ACI = 2 | Intrafamilial difficulties |  |
| ACI = 2 | Problems of trust |  |
| ACI = 1 | Not being able to forget what has been survived |  |
| ACI = 1 | Difficulty to evaluate focus (trauma or social work) |  |
| ACI = 1 | Structural problems in asylum center |  |
| ACI = 1 | Social security: marriage, IV, AHV |  |
| ACI = 1 | Sleep and school attendance is connected |  |
| ACI = 1 | Sans-Papiers: Lack of funding |  |
| ACI = 1 | Lacking awareness regarding health system and required support |  |
| ACI = 1 | Difficulties accepting help |  |
| ACI = 1 | Sans-Papiers: No access to health care / Health insurance |  |
| ACI = 1 | Dependence on third parties |  |
|  | **Resources** |  |
| ACI = 11 | Often strong resilience |  |
| ACI = 8 | Daily structure and activities |  |
| ACI = 6 | Social network |  |
| ACI = 6 | Feeling of being needed (identity) |  |
| ACI = 3 | Acceptance of new life |  |
| ACI = 1 | Following values |  |
| ACI = 1 | Acceptance of help |  |
| ACI = 1 | Religion |  |
| ACI = 1 | Seasonal influence: summer |  |
|  | **Concerns and obstacles towards digital tool** |  |
| ACI = 7 | Relationship and trust building |  |
| ACI = 5 | Lacking sensitivity of digital tool towards emergencies and triggers |  |
| ACI = 5 | Lack of individuality |  |
| ACI = 4 | Recommendation to seek psychotherapy in case of worsening symptoms |  |
| ACI = 2 | Lack of privacy at accommodation |  |
| ACI = 2 | Lack of internet access |  |
| ACI = 2 | Mistrust in app security |  |
| ACI = 2 | Concentration difficulties |  |
| ACI = 1 | IT competency |  |
|  | **Potential use of a digital service** |  |
| ACI = 13 | Bridging tool (preparation, filling, aftercare) |  |
| ACI = 9 | Support for ongoing therapy |  |
| ACI = 8 | Most people have a smartphone, digitalization is future-oriented |  |
| ACI = 8 | Contact person behind the app would be advantageous |  |
| ACI = 7 | No replacement for existing face-to-face services |  |
| ACI = 6 | Tool as source for professionals and relatives of affected |  |
| ACI = 5 | Emergency plan (advice) |  |
| ACI = 5 | Stabilization as primary goal |  |
| ACI = 1 | No regular meetings/safe space can be built |  |
|  | **Digitizable content** |  |
| ACI = 16 | Psychoeducation   1. Psychoeducation on PTSD symptoms (ACI = 8) 2. Sleeping problems (ACI = 4) 3. Stress (ACI = 2) 4. Psychosomatic problems ( ACI = 1)  - Depression (ACI = 1) |  |
| ACI = 14 | Information, explanations on socio-structural everyday aspects/integration:   1. Housing (ACI = 5) 2. Health system (ACI = 5) 3. Occupational Integration (ACI = 5) 4. Contact lists of professionals (ACI = 2) 5. Women’s rights (2)   Swiss culture: punctuality, liability (ACI = 1) |  |
| ACI = 14 | Standardized exercises   1. Body-focused (ACI = 6) 2. Sleep hygiene (ACI = 3) |  |
| ACI = 12 | Activate resources |  |
| ACI = 9 | Connection to social life and activities |  |
| ACI = 7 | Asylum-related legal information |  |
| ACI = 4 | An in-app user-exchange can be helpful |  |
| ACI = 3 | Rules and mediation for in-app user-exchange necessary |  |
| ACI = 3 | Symptom screenings |  |
| ACI = 3 | No imagery exercises |  |
| ACI = 2 | No symptom screenings |  |
| ACI = 2 | Explaining effects of psychotropic drugs |  |
| ACI = 2 | Integration of a feedback/question feature |  |
| ACI = 2 | No trauma exposition elements |  |
| ACI = 2 | Concerns about an in-app user-exchange |  |
| ACI = 1 | Finding patterns of avoidance |  |
| ACI = 1 | Sexual health information |  |
| ACI = 1 | De-normalization of violence |  |
| ACI = 1 | Explanation about alcohol and cigarettes for self-medication |  |
| ACI = 9 | Balance between tailored and generic use |  |
| ACI = 9 | Anonymity: as priority and possibility to reach people |  |
| ACI = 4 | Flexible access: leave, re-enter at any time, incl. offline functionality |  |
| ACI = 3 | No/little push notifications |  |
| ACI = 2 | Fun aspects |  |
| ACI = 1 | Keep updated |  |
| ACI = 1 | Concrete recommendations on the app use |  |
|  | **Advice on the delivery formats** |  |
|  | Simple design and diverse formats:   1. Videos (ACI = 5) 2. Images/illustrations (ACI = 7) 3. Short texts (ACI = 5) 4. Example stories (ACI = 4) 5. Testimonials of other refugees (ACI = 3) 6. Audios (ACI = 2) 7. Metaphors (ACI = 2) |  |
| ACI = 2 | SRC logo as symbol of trust |  |
| ACI = 1 | Avatar as guide through the app |  |
|  | **Advice for psychological language within the tool** |  |
| ACI = 10 | Resource-oriented psychological language: Normalizing, empowering, validating, taking serious, hopeful, patiently, humorous, listening, positive, asking |  |
| ACI = 7 | Avoid stigmatized terms |  |
| ACI = 3 | Consider differences across and within cultures |  |
| ACI = 2 | Often used term: stress |  |
| ACI = 1 | Speak about home and family |  |
| ACI = 1 | Integrate recommendations from medical doctors |  |
|  | **Advice for better accessibility** |  |
| ACI = 9 | Participation of target group in development and dissemination |  |
| ACI = 8 | Access through symptomatology |  |
| ACI = 6 | Access through support of everyday (asylum) life |  |
| ACI = 1 | No focus on symptoms |  |

# **Intervention Adaptation**

- **Criterion 7: Specific treatments elements**
- **Criterion 8: Unspecific elements and therapeutic techniques**

| **Criterion 7: Specific treatment elements**  **Criterion 8: Unspecific elements and therapeutic techniques** | | | | | | | | | | | | | | |
| --- | --- | --- | --- | --- | --- | --- | --- | --- | --- | --- | --- | --- | --- | --- |
| Decision-Nr. | **Treatment elements, techniques, delivery, surface** | **Content** | **Cultural Processes related to mechanism of action** | **Cultural / contextual adaptation** | **Evidence**  e.g., literature review, focus groups, qualitative interview | **Quality of evidence**  Strong  Moderate  Weak | **Decision makers** | | | | | | **State of decision** | |
|  |  |  |  |  | literature  interviews |  | **User Advisory Board** | **Expert group “information”** | **Expert group “Arabic”** | **Expert group “mental health”** | **Beta test** | **Core team, final decision** | pending | made |
| **Language** | |  |  |  |  |  |  |  |  |  |  |  |  |  |
| 1 | Written Arabic language | Levantine Arabic +  The Arabic language is very complex, and the variety of modern standard Arabic is not understood by the broad majority: An easier form of standard Arabic was therefore chosen, called the Levantine Arabic.  A similar dialect of Arabic is usually used for news TV channels in many Arabic-speaking regions. The participants of the expert group “Arabic” originate from different Arabic-speaking countries which allows them to form a broadly understandable form of Levantine Arabic. |  | Add sometimes multiple words to explain, Syrian dialect for recordings was chosen to be most understandable | Literature  Arabic language experts agree on a language that can be understood by most of the people, this includes using sometimes multiple words to describe the same word | moderate | First translations were full of mistakes.  Last versions of each script of each topic was revised, small mistakes were corrected.  If literated, the language is very understandable, Grammar complaints, metaphors often erased | - | Adding synonyms for words that are not understood in every country. | - | Five participants said that the language was clear, understandable and the level was adequate. Two emphasised that even less educated people would understand. Two aprticipants stated that there were a few mistakes in the Arabic translations. | Correct translations without grammatical mistakes are very important.  Use Levantine PLUS additional phrases or words to explain, use a direct, easy, normalising language |  | x |
| 2 | Written Arabic language | Gender: 2 versions: female and male  Even though there already exists a gender-neutral form of the Arabic “you” it is still not used by many people. For our specific target group that should also include many low educated people, the gender-neutral form is not adequate. Even though the male version of Arabic is often used as the “general form” of Arabic, women feel less addressed by the male version. For this reason, it was decided that there have to be two versions of the text in the app: a male and a female version, which can be chosen in the settings. |  |  |  |  | Gender-specific language means respect, we want two versions | agree | agree | agree |  | agree |  | x |
| 3 | Written language basis | German:   - plain language: low complexity, active sentences, avoid nominal style, more verbs, rather short sentences, fewer adjectives, and filler words, prioritise content - Glossary of difficult words and technical terms | Plain language (pre-intermediate, CEFR-A2-level) proof-read by a text agency | A simple and easy basis is necessary for a simple translation for this target group who is often tabooing mental health problems. | Expert interviews  Simple words/sentences, little text, a lot of appreciation, recognition and attention, normalising language, culturally diverse language, take seriously, easy words  Consensus of words to use:   - Think a lot - Worry, worry - nervousness - Hard head - Blocked, my head is full, I just can’t   Consensus of terms not to use:   - Refugee - You are from another country - Patient - Sick, crazy - Victim - Disease - Trauma |  | - | - | agree | - |  | Agree |  | x |
| 3 | Translation process | Arabic:  Multiple step translation:   1. Order a specific translation (incl. document to be translated, and exact costs for the first translation) 2. Format preparation by intercultural counsellor for Arabic translation 3. First written translation/adaption by a professional Arabic-German translator to a written standard Arabic 4. Proof-reading and commenting by “expert group Arabic”, mainly focusing on wording, adding synonyms, different versions of translations 5. Integration and decision on all Arabic comments through intercultural counsellor with a main focus on understandability and elimination of technical terms 6. Content-proof-reading by User advisory board (see next step) 7. Finalisation of the version 8. Translation to female version of the translation and coarse proof-reading by intercultural counsellor (incl. clerical errors) | As the Arabic language is so complex and words are so region-specific one person alone could not translate correctly. An elaborated multiple-step translation is therefore needed in order to reach a maximum of simplicity and correctness. | In critical cases, multiple synonyms were used |  |  | Remarks especially in the beginning of the meetings  Revised texts are very well written. | - | agree | - |  | agree |  | x |
| 4 | Process of writing content scripts in German | 1. Conceptualisation of one specific topic 2. Discussion with mental health experts, expert group “information”, other individual experts 3. Revision of concept and writing of content 4. Content proof by corresponding experts 5. Revision and integration of feedback and preparation for translation 6. Translation process (see previous step) 7. Review by User Advisory Board focusing on the content, along with design 8. Clarification and integration of questions/comments of the User Advisory Board | Feedback-loops with user advisory board | Specific changes or recommendations are documented below for each chapter |  |  | agree | agree | agree | agree |  | agree |  | x |
| **Peer support / guidance** | |  |  |  |  |  |  |  |  |  |  |  |  |  |
| 5 | Using Support | Peer Guidance:  Arabic speaking people living in Switzerland will be trained and will then be supporting participants using the app. They will be supervised by an Arabic speaking psychotherapist living in Germany.  They send one text message weekly and can answer text messages within 3 working days. | Based on evidence guided e-mental health interventions show higher effects compared to unguided interventions.  Requirements:  Peers are Arabic speaking, males or females, can speak German on an advanced level and are motivated to support participants. |  | Peer-guided e-mental health interventions appear to have an additional effect on the primary outcomes  (Berger, 2017; Berger, Caspar, et al., 2011; Berger et al., 2014; Berger, Hämmerli, et al., 2011; Bur et al., 2022; Schulz et al., 2016)  Expert interviews  Psychotherapy cannot be replace, tool cannot stand alone, app could accompany an ongoing psychotherapy, would suggest guidance  Peer exchange on mental health could be helpful  Give the opportunity to interact, give feedback  Chat/forum: clear rules, moderation  Contact person is important, maybe individually selectable | strong | Behind the app there should be a contact person that answers individual questions. | - | - | - | Five participants wished to receive 1-3 messages per week and a quick response to questions. Two participants said that a response to questions is fine within 2-3 days, one would prefer a daily answer, another within 6-7 hours.  Both peers said that one message per week is not enough and the response time should be shortened. Technical issues should be resolved within 48 hours. | We train people who have themselves a migration background, speak Arabic and German (for better communication with us)  Reduce answering time from 72 hours to 48 hours. |  | x |
| 6 | Form of support | Training of peers:   1. Getting to know the Sui app 2. Getting to know set text templates 3. Getting to know ways of interacting, such as: active listening, paraphrasing, validating of suffering, normalizing psychological symptoms 4. Learning to suggest sub-chapters of the app 5. Getting to know area of responsibility and boundaries | Importantly, peers need to set boundaries, because they cannot help with the practical realisation of problem solving | Peers have a similar background | Based on learnings from other interventions that have been evaluated in (Berger, Hämmerli, et al., 2011; Harper Shehadeh et al., 2020; Mediavilla et al., 2022; Seewer et al., 2024; Spaaij et al., 2022) |  | Don’t use nationality and last names.  First name, gender is important, and that the person has expertise with the rights in Switzerland. | - | - | - | Some participants said peers should be able to answer technical issues with the app.  One person said the peer should talk about their experiences in Switzerland.  Three wished for personal information about the peer person, such as age, country of origin, duration of living in Switzerland, canton of residence.  Receiving responses to individual questions was perceived motivating by one participant.  Three participants said that this support will lead to a n enhanced use of the app.  One said, the peer should give tips about useful contacts in Switzerland.  Another one said that the active messages from the peer are good to have an idea on what to ask.  Another one said the messages should not be too long.  Beta test peers feedback:  One peer said that exercises should be linked in the messages, and also images and videos should be sharable.  One peer said that she would like to share details about herself.  One peer said that one participant had not received the push notification from a new message.  Both peers agreed that the message templates sounded too formal.  One peer said that the main questions revolved around technical issues.  Both peers said that it is important to create a community feeling for the participants for them to not feel like they are alone. | The training includes getting to know the Sui app, the technical working platforms, using text templates  After the beta test: including a personal presentation of the peer at the beginning of the conversation.  The message templates were revised and made less formal.  We include more FAQs about technical issues. |  | x |
| **Chapter “general”** | |  |  |  |  |  |  |  |  |  |  |  |  |  |
| 1 | Introductory session | Self-help interventions seem to work well, if they are built modular with clear instructions.  Because the Sui app is a service for very heterogenous needs, all sessions are unlocked from the beginning. For this reason, an introduction module is necessary. | - | - | Expert interviews | moderate | - | - | - | The many chapters can seem overwhelming, an introduction would help |  | The heterogeneity of the target group does not allow a predefined modular structure, we insert a general introduction chapter |  | x |
| 2 | Feedback session | Users should get the possibility to give feedback on the app. | - | - | Expert interviews | strong | agree | agree | agree | agree |  | agree |  | x |
| 3 | Sui’s neighbourhood, back story | Profiles of the protagonists of the Sui app | - | - | - | - | Showing examples of others that succeeded could be helpful | - | - | People tend to like hearing stories, even dramatic descriptions of fictive stories |  | The users should get the profiles of the protagonists to know with whom they can identify. |  | x |
| **Chapter**  **“information”** | |  |  |  |  |  |  |  |  |  | |  |  |  |
| 1 | Socio-structural elements: Everyday life support as a main category in the app | Topics in the information chapter are the following post-migration factors:   - Housing - Health system - Health tips - Finances - Asylum process - Residence status - Family reunion - Social integration - Work & education - Emergency contacts | First agenda to solve mental issues are often to reduce everyday life struggles. | Asylum-relevant Swiss-specific information was added to support  administrative self-help | Literature review: Dropout in other interventions, main mental health support hurdles  ADAPT model (Silove, 2013)  Ecological model of refugee distress (K. E. Miller & Rasmussen, 2017)  Difficulties in social integration (Schick et al., 2016)  PMLD and symptom reduction (Djelantik et al., 2020)  Further literature :  (Byrow et al., 2020; Campbell, 2018; Gleeson et al., 2020; Hajak et al., 2021; Jannesari et al., 2020; Nickerson et al., 2011; Porter & Haslam, 2005)  Expert interviews  State that in most of the cases asylum and living related issues have to be addressed first in therapy/dialogue, re-read first information from asylum center  Main obstacles for recovey: asylum situation, search for housing, jobs, daily structure, language difficulties, dependency on third parties, intrafamilial problems (e.g. violence)  Digitizable: links, pool of laypersons, reference to professional help and communities, activities, copting with everday life, Swiss cultural information, social counselling, search for housing, insurance system, information on returning home, on the health system, waste disposal system, specialists offices, school system, adult education, part-time jobs, public transportation, asylum hearing, information specific to the canton, revocation of diplomas  Target group interviews  Family reunification complicities, language, work, work at older age, work with higher education, (work and) uncertain residency status, negative asylum decision, discrimination, rules/laws, family problems, dependency on third parties, revocation of diplomas, wish for (social) support for administrative issues/key persons | strong | These are of the most relevant hurdles in our lives.  One says: panic attacks only started after receiving the asylum decision.  Guidance on rights about every topic. | Could be expanded with more relevant topics, but contain the most asked for | agree | In therapy, dialogue, and counselling sessions these are the topics first asked about | All participants agreed that the app’s content is very helpful. Especially “social integration” was perceived positive among five participants. One person said that he is disappointed, because he did not learn how to socially integrate through the chapter. Half of the participants found “housing” and “health system” supportive.  Four participants wished for a chapter about the “asylum process” (was not included yet).  There were other suggestions from different participants: scholarships, education, history of Switzerland, drugs, forced marriage and old-age provision. | agree  Add contact details for every topic – right form of doing that was difficult, added later in pdf files.  The chapter “asylum procedure” is added to the app after the beta test, because of time reasons. |  | x |
| 2 | Delivery method | Text-based information |  |  |  |  | Short texts can be useful, because not everybody dares to contact someone. |  |  |  |  |  |  | x |
| 3 | Delivery method | Contact details for specific topics |  |  |  |  | Many people, especially elderly people cannot read well and rather call someone. |  |  |  |  |  |  | x |
| **Session “housing”** | |  |  |  |  |  |  |  |  |  |  |  |  |  |
| 1 | housing | Goal: Where and especially how to apply for an apartment, tips for the application process, what to look out for when moving in/out and in renting contracts, what aspects are important for living in Switzerland, what obligations renting an apartment entails, where to get support |  |  | Expert and target group interviews | strong | Chapter very understandable. Order of the modules very clear. | The content was based on a document from the Swiss Red Cross Cantonal Association of Bern from the real estate and property service.  Explain additional costs: Net rent  Service charges  Serafe  Electricity costs  Liability insurance  Rental deposit (deposit or insurance) |  |  |  |  |  | x |
| 2 | housing | Searching for a home |  |  | Expert and target group interviews | strong | Everything good, make hope in the end of the chapter to find an apartment even without having the support of acquaintances. |  |  |  |  |  |  | x |
| 3 | housing | Application for a home |  |  | Expert and target group interviews | strong | Everything important is included.  Templates are useful. | Form is often distributed when visiting the apartment, explain to ask there for it |  |  |  | Template of one of our protagonists. Now inserted as text or picture, because pdfs are not technically possible yet. |  | x |
| 4 | housing | Renting a home |  |  | Expert and target group interviews | strong | Everything clear. | Control contract: correct name, correct renting date, check notice periods. |  |  |  |  |  | x |
| 5 | housing | Living together with the neighbourhood |  |  | Expert and target group interviews |  | Nothing to change. |  |  |  |  |  |  | x |
| **Note: This is an abbreviated version. The complete RECAPT documentation for the Sui app can be requested:* [*rilana.stoeckli@unibe.ch*](mailto:rilana.stoeckli@unibe.ch) | | | | | | | | | | | | | | |
| **Chapter**  **“well-being”** | |  |  |  |  |  |  |  |  |  |  |  |  |  |
| 1 | Early psychological support: Address  mental health issues at symptom level, as a second main category | Psychological symptoms:   - Stress - Sleep - Boredom, inactivity, loneliness, emptiness - Chronic pain - Emotions: Fear, anger, mistrust, anxiety - (Alcohol and drug abuse)   Further, but excluded:   - Dissociation | These symptoms are accessible and are also named by the target group.  A very first prototype containing mainly PTSD-psychoeducation was beforehand rejected by the User Advisory Board. | Well-being as a second main category to address the most named symptoms | Literature review:  (Borho et al., 2021; Hassan et al., 2015)  Expert interviews  approach via symptomatology, help to reach self-efficacy  provide information on various acute topics as a preparation for therapy  symptoms: sleep disturbances, concentrationconcentratees, nervousness/restlessness, physical complaints/psychosomatics, difficulties of social integration, loneliness, anxiety, feelings of guilt, lowered trust, lowered self-esteem  Target group interviews  Stress from experiences, physical complaints since escape/psychosomatics, worrying/longing about the ones left behind, feelings of guilt, pressure in language school, not being able to forget experiences, occupying thoughts, shame about health problems, loss of status, not feel like a full member of society, wish for (social) support for talking/key persons, recognition of missing family members | strong | Agree, mention panic attacks, feelings of pressure, hopelessness and more | - | - | Agree and more, such as ambiguous loss, grief, substance use | Three participants found “sleep” helpful. Two participants each found “stress” and “chronic pain” useful. The written exercises included in the “sleep” chapter were rated as superficial by three participants. | Agree, implement more later, provide contact information of emergency services, we added information that not all exercises will work for everyone. |  | x |
| 2 | Address mental health issues with various techniques | Topics of well-being:   - Psychoeducation - Audio Exercises: body therapy, hypnotherapy, trauma-sensitive yoga, breathing, mindfulness - Problem solving strategy - Values compass - Activity plan (behavioural activation) - Sleep hygiene | Based on therapy experiences various techniques work on this heterogenous target group | A variety of techniques are offered in the app to respond to the heterogeneity of the target group | Literature  Expert interviews:  Digitizable: pyschoeducation: description of stress, clinical pictures, self-meication, psychosomatics, course of PTSD, dealing with nightmares, sleeping problems, symptoms, psychopharmaceutic, sexual health, effectiveness of treatments  Breathing exercises, relaxation exercises, treatment of symptoms (e.g. sleep hygiene), physical exercises, values compass, diary, acceptance content | strong | There has to be something for well educated people as well as low educated people, the content has to be appealing | - | - | Not everything works for everybody, we experience a broad heterogeneity in preferred exercises |  |  |  | x |
| 3 |  | - lifeline |  |  | Literature  Expert interviews | moderate | Lifeline: can be triggering, very clear instructions needed | - | - | Lifeline: was successful in another small study  Needs a lot of attention in case of triggering |  | Lifeline: will not be included because technically too complex at the moment |  | x |
| 4 | Psychoeducation | - Stress - Sleep hygiene - Chronic pain - emotions |  |  | Expert interviews  Stigma: not wanting to be “crazy”, shame, difficulties expressing oneself, need for normality |  |  |  |  |  |  |  |  | x |
| 5 | Body-focused exercises | - body therapy - hypnotherapy - trauma-sensitive yoga - breathing exercises - mindfulness |  |  | Expert interviews  Explain effect of exercises  Good experiences with body-related exercises |  | They cannot help in crises, can help to relax | - | - | Successful in other psychotherapies |  | Repeat that not all exercises help everybody, and repetition and time is important, avoid releasing feelings of failure and expectations, try to sound inviting not commanding |  |  |
| 6 | Problem solving | Stop, slow down, think & continue:   - skills training - regulating emotions |  |  | Literature: (Bur et al., 2022; Nezu et al., 2012), Doing What Matters in Times of Stress (Acarturk et al., 2022; Epping-Jordan et al., 2016; Tol et al., 2020; World Health Organization, 2020) |  |  |  |  |  |  | Problem solving strategy with a focus on “stop” and “slow down” to regulate intensive emotions |  | x |
| 7 | Behavioural, resources activation | - planning activities - thankfulness - activation of strength sources - values - staying present |  |  |  |  |  |  |  |  |  |  |  |  |
| 88 | Handling emergencies | At the beginning of the app use every participant reads a disclaimer that the app is not adequate for emergency situations.  Within the app there is one session that includes the most common emergency contact details. |  | Explanation of the uniforms of emergency services, | Expert interviews  Trigger moments cannot be intercepted  People might get triggered by uniforms of emergency services; a heads-up needed. | strong | “I want to know what to do in case of a panic attack” |  | - | Disclaimer at the beginning necessary, exclusion criteria  One problem is that even national emergency services often cannot communicate with the people because of language barriers. |  | Disclaimer, exclusion criteria for RCT participation, emergency information |  | x |
| **Session “stress”** | |  |  |  |  |  |  |  |  |  |  |  |  |  |
| 1 | Normalisation  psychoeducation | **Intro stress**  Goal: To get as close as possible to the target group's everyday life, to show that stress is quite normal in their situation.  *«Hamid ist froh, dass er nicht mehr in der Kollektivunterkunft leben muss. Dort konnte er nicht gut schlafen, weil er das Zimmer mit drei anderen Männern teilen musste. Diese Zeit im Zentrum war für ihn sehr belastend. Er musste oft an seine Heimat und Familie denken und konnte überhaupt nicht verstehen, warum der Asylprozess so lange dauerte.»*  Goal: To create understanding that you can reduce stress yourself and thus better manage everyday life and be there more for family members.  Partly based on the Doing What Matters in Times of Stress guide from the WHO (Epping-Jordan et al., 2016) |  |  | Literature (Alemi et al., 2014; Al-Smadi et al., 2017)  Expert interviews  psychoeducation on stress would be easily digitizable  Target group names “stress” a lot as a symptom | strong | To understand vicious circles of e.g., a panic attack can help to understand what is normal.  It's written exactly how a doctor would explain it. It feels like I feel when I am stressed and it shows me that it’s normal.  The first used metaphor that stress is like an “alarm system” is not understandable, it reminds of the alarm clock at school.  Stress used to be a taboo to speak about in the past, but since the conflict it has become commonplace. It can be expressed as depression, inattentiveness, sadness, always thinking about it. It can occur in every situation.  Still, often, we don’t speak about stress in front of father or husband in fear of getting excluded, suppressed or because of shame, fear of not being taken seriously, fear of the past.  The common answer “Alhamdulillah" is still widely spread to answer “how are you?”  Other words that can be used are “I am psychologically tired, angry, nervous”.  It can cause inattentiveness, sleeping disorders, worries, isolation, listlessness, forgetfulness, psychological pressure, fast heart beating, diabetes, dizziness, pale face, rash, headache, high blood pressure.  Common tips against stress are: distraction, doing sports, writing, thinking about beautiful memories, listening to music, dancing in a group, getting up early, doing a job/activity if possible, be with friends, reading a book, discuss it with somebody, go to a specialist, do hobbies  People who have stress should find the sources of it, set the goal to get away from the stress, get in contact with others, be motivated to help others, do voluntary work, focus problems of others, write, do gardening | - |  | Explaining that the new situation in Switzerland and the related strains affect the body with stress, but in this app you can learn skills to deal with it. | The alarm system sounds a bit technical, is it understandable for the target group? | The metaphor of the alarm system is removed, instead the system regulating stress in our brain is described as a ”regulating system” |  | x |
| **Note: This is an abbreviated version. The complete RECAPT documentation for the Sui app can be requested:* [*rilana.stoeckli@unibe.ch*](mailto:rilana.stoeckli@unibe.ch) | | | | | | | | | | | | | | |

- **Criterion 9: Surface adaptations**

| **Criterion 9: Surface adaptions** | | | | | | | | | | | | | | |
| --- | --- | --- | --- | --- | --- | --- | --- | --- | --- | --- | --- | --- | --- | --- |
| **Decision-Nr.** | **Treatment elements, techniques, delivery, surface** | **Content** | **Cultural Processes related to mechanism of action** | **Cultural / contextual adaptation** | **Evidence**  e.g., literature review, focus groups, qualitative interview | **Quality of evidence**  Strong  Moderate  Weak | **Decision makers** | | | | | | **State of decision** | |
|  |  |  |  |  | literature  interviews |  | **User Advisory Board** | **Expert group “information”** | **Expert group “Arabic”** | **Expert group “mental health”** | **Beta test** | **Core team, final decision** | **pending** | **made** |
| **Technical decisions** | | | | | | | | | | | | | | |
| 1 | Overall delivery method | Native app | No changes | No changes | Expert interviews  Smartphones are widespread within the target group, internet is important to the target group, new interventions should be future oriented, help to reduce stigma and can be helpful if low-threshold, must always be up-to-date  A native app is to be preferred to a progressive web app (PWA), because PWA are not easily usable on iOS.  We developed on the existing platform produced by the Freie Universität Berlin, where other Arabic interventions were developed. Benefiting from this expertise is highly valuable, since the Arabic language is tricky when it comes to IT.  Benefits of a digital approach: more people can be reached and earlier reached, information can be given pre-therapy, anonymity guaranteed, quickly share information in various languages, can be fun  Disadvantages: no relationship building, psychotherapy cannot be replaced, concern of suicidal emergencies, triggers cannot be intercepted, no sensitivity, loss of reality  Target group interviews  Might be helpful, if anonymously, open to try | strong | agree | agree | agree | agree | All participants complained about the long loading times of the app. Half of the participants reported that links did not work and some stated that the app crashed because of non-working links. | Agree, loading times, and reasons for crashes are prioritised in technical improvements, links are controlled. |  | x |
| 2 | Development approach | Agile, incremental, bottom-up development, design-thinking, user-centered | User advisory board meetings with translator | Participatory approach | Expert interviews  Work close to the target group, incrementally | Strong | Agree | Agree | Agree | Agree |  | Agree |  | x |
| 3 | Delivery methods | - Text - Illustrations (partly animated) - Explanation Videos - Testimonial videos - Audio exercises - Dubbing of text (planned for after the study) - List exercises, keyword input - Links to contacts | Various input sources are appropriate for this heterogenous group | Simple language, intuitive illustrations, easy audios, links to support | Expert interviews  Way of using the app should be customisable  Stay general, use videos, audios, little text, low irritation, give the opportunity to try out new things  The handling should be self-explanatory | Strong | Short, fundamental information, solutions and answers.  Information should be supported with creative illustrations. | Agree | Agree | Agree | Add additional links to websites and information about authorities. | Agree, links are controlled and added where suitable. |  | x |
| 4 | Motivation | Gamification | No changes |  |  |  | e.g. use tailoring elements as rewards (e.g. choosing colours) | agree | agree | agree |  | Active gamification needs more resources that we have, we postpone | x |  |
| 5 | Tailoring | Filter options |  |  |  |  |  |  |  |  | All participants agreed that the structure of the app content is logical and easy to understand. | Decide to not filter from the beginning to provide all available information and rather filter on topic-level if desired. | x |  |
| **Surface** | |  |  |  |  |  |  |  |  |  |  |  |  |  |
| 1 | Design agency selection | Illustrations of on the topics of family reunion and stress of two agencies were presented to the core team and the User Advisory Board. | The agency with the more realistic depictions of people was selected. Abstract art was not perceived as adequate. |  |  |  |  |  |  |  |  |  |  | x |
| 2 | Illustrations | Illustration should support information to make it more visualized, accompanied by examples.  Simple but quite realistic images, abstract illustrations are not understandable. | Feedback-loop with designers and user advisory board | No abstract illustration used, colofrul, asylum-related everyday life illustrations |  | Strong | Illustration style close to reality, people not too happy, not too sad either, adapted for situations, be careful for cultural and racist delicacies, not to much in one image. | agree | agree | agree | All participants found the illustrations beautiful and suitable for the content. One said that the illustrations support the content. The colours were rated as neutral and nice.  Two said that they would have preferred the dark-mode. | Agree, except for offering to change to dark-mode because this technical development is too costly |  | x |
| 3 | Illustration process | 1. Description of the desired illustrative display within the scripts 2. Creation of graphic B/W sketches 3. Review of sketches by User Advisory Board together with step 7 of the process o topic scripts 4. Adaptation of sketches and detailed realisation to colourful vector illustrations 5. Last review of vector illustrations by the core team 6. Last revision and release |  |  |  |  |  |  |  |  |  |  |  |  |
| 4 | “Avatar” | The bird Sui was chosen as the app companion, it was not perceived as offensive or disruptive to anyone. | Feedback-loop with designers and user advisory board | Neutral, widely known animal that has no “bad” connection | Expert interviews  Use animals, examples, and stories | strong | Something like an avatar should guide you through the app and motivate to do things and explain how to do things.  The bird is pleasing, no bad emotions attached to it. | agree | agree | agree | Two emphasised that the Sui bird is nice, | Decided on a bird instead of a human or any other animal representation of an avatar to avoid being exclusive. |  | x |
| 5 | Storyline, personas | Multicultural neighbourhood living in a apartment block somewhere in Switzerland. People originating from some of the largest asylum-groups in Switzerland and various life stories:   - Hamid, 22, Afghanistan - [Bisrat, 35, Eritrea   Merhawit, 4, Eritrea]   - Amir, 37, Syria - [Nuriye, 45, Turkey   Umut, 50, Turkey  Hiwa, 17, Turkey]   - [Anutas, 35, Sri Lanka   Suvetha, 32, Sri Lanka  Thinesh, 6, Sri Lanka]   - Nadia, 22, Switzerland - Henriette, 85, Switzerland |  | In order to represent various cultures and people with different stories living in Switzerland a neighbourhood living in an apartment block together was chosen. The descriptions and the illustrations of each person was revised by at least 3 representatives of the corresponding country origin and then adapted based on the feedback. |  |  |  |  |  |  | They were perceived as reality-related. Three participants said that the people were very beautiful and could identify well with them.  Two participants said that the bodies of the people did not reflect reality, they were either too fat or too slim.  The different skin colours were perceived positive by two participants. |  |  |  |
| 6 | Session intros | Each session starts with an introduction module to clarify |  |  |  |  |  |  |  |  |  |  |  |  |
| 7 | Target group | Arabic-speaking adults  Rather low level of education  Less than five years in Switzerland | ? | First relevant topics when arriving new to Switzerland | Literature review  Expert and target group interviews  Expert interviews  Might be helpful for relatives as well | strong | The chosen topics are not very interesting for people who have been living in Switzerland for a long time. They would have been interesting mainly in the beginning. | Important topics that always come up when working with migrants/refugees | agree | agree |  | The choice of topics is a combination of everyday life topics related to asylum and symptom-based topics |  | x |
| **Audios** | |  |  |  |  |  |  |  |  |  |  |  |  |  |
| 1 | Audio Arabic | Audio exercises:   - Two versions of each audio: one addressing females and one addressing males, like the written text   Two voices, a man and a woman, 50:50 |  |  | Literature | moderate | It does not matter if the voice of the exercises is a male or female voice.  The quality of the audio (sound quality) is important to be high. | - |  | Women might be triggered by a male voice, clarify with target group | The audio exercises were valued by half of the participants. | Decide to use a male and female voice equally often |  | x |
| 2 | Audio Arabic | Decision for Arabic voice |  |  |  | strong | Democratic choice of two voices (one male, one female) out of 5 male and 4 female voices | . | Democratic choice of two voices (one male, one female) out of 5 male and 4 female voices |  |  | Decision for one female Syrian voice, one male Syrian voice |  | x |
| 3 | Delivery method | Exercises from the trauma sensitive yoga (TSY®) program in Switzerland, experience with migrants/refugees  More exercises from a body therapist from the outpatient clinic of the SRC (for war and torture survivors)  One exercise from a hypnotherapist, experience with migrants/refugees  One exercise from a previous internet intervention |  |  | Expert interviews  Literature |  | An exercise within a video can be distracting, animations would be better to stay concentrated, but to also understand how the exercise is done.  Music in the background has a relaxing effect | - | - | agree | There should be more illustrations for the exercises to be able to understand the correct postures. | Decide to use illustrated pictures, because animated illustration would be too costly.  Decide to use a underlying continuous background sound and nature sounds for longer breathing breaks. |  | x |
| **Videos** | |  |  |  |  |  |  |  |  |  |  |  |  |  |
| 1 | Videos | Testimonials of people similar to the target group for most of the topics. |  |  | Expert interviews  testimonials of peers could be supportive, success stories |  | Agree to participate in the video production | agree | agree | - |  | agree |  | x |

# **Measuring Outcomes**

- **Criterion 10: Questionnaires and clinical interviews**
- **Criterion 11: Implementation measures**

| **Criterion 10: Questionnaires and clinical interviews** | | | |
| --- | --- | --- | --- |
| **Category** | **Instruments used for outcome assessments** | **Translation / validation / adaptation** | **Sources** |
| **Preparation phase** |  |  |  |
| Interview guideline for asylum care interviewees | - Introduction of the rough project idea - First thoughts on the general project idea - Potential relief for experts - Psychological problems in target group - Main barriers for recovery - Resources in target group - Digitizable content for a support tool - General estimation on the effectiveness of a digital support tool - Terms to use/not to use with target group - Known/used tools - General remarks | Interviews were held in German, codes in the analyses were translated to English |  |
| Interview guideline for intercultural interpreters | *Same/similar interview guideline as for asylum care interviewees* | Interviews were held in German, codes in the analyses were translated to English |  |
| Interview guideline for target group interviewees | - Introduction of the rough project idea - Short presentation on person, age, profession, living situation - Duration of living in Switzerland - Life priorities, proudest achievements - Well-being, indicators - Well-being in Switzerland, everyday life - Challenges, daily struggles, main struggles, worsening factors - Problem management in home country - Support seeking behaviour in Switzerland - Needs for support, desired support - Barriers to seek help - Recommendations for newly arrived refugees - Life situation of family - General remarks | Interviews were held in German and Arabic and were translated by an intercultural interpreter. The transcripts were written in German and codes in the analyses were translated to English. |  |
| **Development phase** |  |  |  |
| User Experience (UX) Test | Open questions (Think Aloud): Which information do you find in the app? Why was the app created and for whom?  UX tasks:   - General use of the app, incl. language change, login/logout, saving as favourite, request help, - Find content, incl. descriptions of Sui/neighbourhood people, concrete exercises, tips for sleeping, tips for housing applications, help with invoices, emergency numbers, help with labour rights, stress information, asylum process information | The test users were first asked the open questions to which they could answer openly.  For the second part the test users were instructed to perform specific tasks within the app to evaluate its usability and user experiences. The participants were encouraged to think aloud, provide feedback, and maybe ask for hints, as they completed each task to help identify usability issues or areas for improvement. Their feedback and their app interactions were noted by an observer. |  |
| Interview guideline for beta test users | Questions about:   - Functionality and structure - Visual appearance - Content (positive, negative, missing) - Identification with the Sui neighbourhood characters - Peer support (identifying what was helpful, not helpful) - Adherence (motivation, frequency) - Use of the app in everyday life | The questions were asked in a semi-structured interview in German and translated to Arabic. |  |
| Interview guideline for beta test peers | Questions about:   - Personal experience (feelings, users’ concerns, usefulness of support, interaction) - Support (scope, advantages, disadvantages, optimal support) - Process and workflow (training, time spent, response time, expectations, support with difficult questions, supervision) | The questions were asked in a semi-structured interview in German. |  |
| **Planned assessments for the Sui RCT** |  |  |  |
| Self-assessment  **Primary outcome** | Quality of life on 4 domains  WHOQOL-BREF  26 items | Validated (tested in Kuwait) translated Arabic version | (Ohaeri & Awadalla, 2009; Skevington et al., 2004; WHOQOL-Group, 1998) |
| **Note: This is an abbreviated version. The complete RECAPT documentation for the Sui app can be requested:* [*rilana.stoeckli@unibe.ch*](mailto:rilana.stoeckli@unibe.ch) | | | |

# References

Aarethun, V., Sandal, G. M., Guribye, E., Markova, V., & Bye, H. H. (2021). Explanatory models and help-seeking for symptoms of PTSD and depression among Syrian refugees. *Social Science & Medicine*, *277*, 113889. https://doi.org/10.1016/j.socscimed.2021.113889

Acarturk, C., Kurt, G., Ilkkursun, Z., Uygun, E., & Karaoglan-Kahilogullari, A. (2022). “Doing What Matters in Times of Stress” to Decrease Psychological Distress During COVID-19: A Randomised Controlled Pilot Trial. *Intervention*, *20*(2), 170. https://doi.org/10.4103/intv.intv_29_21

Al Masri, F., Müller, M., Nebl, J., Greupner, T., Hahn, A., & Straka, D. (2021). Quality of life among Syrian refugees in Germany: A cross-sectional pilot study. *Archives of Public Health*, *79*(1), 213. https://doi.org/10.1186/s13690-021-00745-7

Alemi, Q., James, S., Cruz, R., Zepeda, V., & Racadio, M. (2014). Psychological Distress in Afghan Refugees: A Mixed-Method Systematic Review. *Journal of Immigrant and Minority Health*, *16*(6), 1247–1261. https://doi.org/10.1007/s10903-013-9861-1

Al-Smadi, A. M., Tawalbeh, L. I., Gammoh, O. S., Ashour, A. F., Alshraifeen, A., & Gougazeh, Y. M. (2017). Anxiety, stress, and quality of life among Iraqi refugees in Jordan: A cross sectional survey: Quality of life among refugees. *Nursing & Health Sciences*, *19*(1), 100–104. https://doi.org/10.1111/nhs.12323

Aragona, M., Pucci, D., Mazzetti, M., & Geraci, S. (2012). Post-migration living difficulties as a significant risk factor for PTSD in immigrants: A primary care study. *Italian Journal of Public Health*, *9*(3). https://doi.org/10.2427/7525

Bartolomei, J., Baeriswyl-Cottin, R., Framorando, D., Kasina, F., Premand, N., Eytan, A., & Khazaal, Y. (2016). What are the barriers to access to mental healthcare and the primary needs of asylum seekers? A survey of mental health caregivers and primary care workers. *BMC Psychiatry*, *16*(1), 16–336. https://doi.org/10.1186/s12888-016-1048-6

Berger, T. (2017). The therapeutic alliance in internet interventions: A narrative review and suggestions for future research. *Psychotherapy Research*, *27*(5), 511–524. https://doi.org/10.1080/10503307.2015.1119908

Berger, T., Boettcher, J., & Caspar, F. (2014). Internet-based guided self-help for several anxiety disorders: A randomized controlled trial comparing a tailored with a standardized disorder-specific approach. *Psychotherapy*, *51*(2), 207–219. https://doi.org/10.1037/a0032527

Berger, T., Caspar, F., Richardson, R., Kneubühler, B., Sutter, D., & Andersson, G. (2011). Internet-based treatment of social phobia: A randomized controlled trial comparing unguided with two types of guided self-help. *Behaviour Research and Therapy*, *49*(3), 158–169. https://doi.org/10.1016/j.brat.2010.12.007

Berger, T., Hämmerli, K., Gubser, N., Andersson, G., & Caspar, F. (2011). Internet-Based Treatment of Depression: A Randomized Controlled Trial Comparing Guided with Unguided Self-Help. *Cognitive Behaviour Therapy*, *40*(4), 251–266. https://doi.org/10.1080/16506073.2011.616531

Blackmore, R., Boyle, J. A., Fazel, M., Ranasinha, S., Gray, K. M., Fitzgerald, G., Misso, M., & Gibson-Helm, M. (2020). The prevalence of mental illness in refugees and asylum seekers: A systematic review and meta-analysis. *PLOS Medicine*, *17*(9), e1003337. https://doi.org/10.1371/journal.pmed.1003337

Borho, A., Morawa, E., Schmitt, G. M., & Erim, Y. (2021). Somatic distress among Syrian refugees with residence permission in Germany: Analysis of a cross-sectional register-based study. *BMC Public Health*, *21*(1), 896. https://doi.org/10.1186/s12889-021-10731-x

Bur, O. T., Krieger, T., Moritz, S., Klein, J. P., & Berger, T. (2022). Optimizing the context of support of web-based self-help in individuals with mild to moderate depressive symptoms: A randomized full factorial trial. *Behaviour Research and Therapy*, *152*, 104070. https://doi.org/10.1016/j.brat.2022.104070

Byrow, Y., Pajak, R., Specker, P., & Nickerson, A. (2020). Perceptions of mental health and perceived barriers to mental health help-seeking amongst refugees: A systematic review. *Clinical Psychology Review*, *75*, 101812. https://doi.org/10.1016/j.cpr.2019.101812

Campbell, M. R. (2018). Social determinants of emotional well-being in new refugees in the UK. *Public Health*, *164*, 72–81. https://doi.org/10.1016/j.puhe.2018.07.022

Correa-Velez, I., Gifford, S. M., & Barnett, A. G. (2010). Longing to belong: Social inclusion and wellbeing among youth with refugee backgrounds in the first three years in Melbourne, Australia. *Social Science & Medicine*, *71*(8), 1399–1408. https://doi.org/10.1016/j.socscimed.2010.07.018

Djelantik, A. A. A. M. J., de Heus, A., Kuiper, D., Kleber, R. J., Boelen, P. A., & Smid, G. E. (2020). Post-Migration Stressors and Their Association With Symptom Reduction and Non-Completion During Treatment for Traumatic Grief in Refugees. *Frontiers in Psychiatry*, *11*, 407. https://doi.org/10.3389/fpsyt.2020.00407

Drescher, A., Kiselev, N., Akhtar, A., Acarturk, C., Bryant, R. A., Ilkkursun, Z., von Känel, R., Miller, K. E., Pfaltz, M. C., Schick, M., Schnyder, U., Sijbrandij, M., Spaaij, J., & Morina, N. (2021). Problems after flight: Understanding and comparing Syrians’ perspectives in the Middle East and Europe. *BMC Public Health*, *21*(1), 717. https://doi.org/10.1186/s12889-021-10498-1

Epping-Jordan, J. E., Harris, R., Brown, F. L., Carswell, K., Foley, C., García-Moreno, C., Kogan, C., & van Ommeren, M. (2016). Self-Help Plus (SH+): A new WHO stress management package. *World Psychiatry*, *15*(3), 295–296. https://doi.org/10.1002/wps.20355

Farahani, H., Joubert, N., Anand, J. C., Toikko, T., & Tavakol, M. (2021). A Systematic Review of the Protective and Risk Factors Influencing the Mental Health of Forced Migrants: Implications for Sustainable Intercultural Mental Health Practice. *Social Sciences*, *10*(9), 334. https://doi.org/10.3390/socsci10090334

Gleeson, C., Frost, R., Sherwood, L., Shevlin, M., Hyland, P., Halpin, R., Murphy, J., & Silove, D. (2020). Post-migration factors and mental health outcomes in asylum-seeking and refugee populations: A systematic review. *European Journal of Psychotraumatology*, *11*(1), 1793567. https://doi.org/10.1080/20008198.2020.1793567

Hajak, V. L., Sardana, S., Verdeli, H., & Grimm, S. (2021). A Systematic Review of Factors Affecting Mental Health and Well-Being of Asylum Seekers and Refugees in Germany. *Frontiers in Psychiatry*, *12*, 643704. https://doi.org/10.3389/fpsyt.2021.643704

Harper Shehadeh, M. J., Abi Ramia, J., Cuijpers, P., El Chammay, R., Heim, E., Kheir, W., Saeed, K., van Ommeren, M., van’t Hof, E., Watts, S., Wenger, A., Zoghbi, E., & Carswell, K. (2020). Step-by-Step, an E-Mental Health Intervention for Depression: A Mixed Methods Pilot Study From Lebanon. *Frontiers in Psychiatry*, *10*, 986. https://doi.org/10.3389/fpsyt.2019.00986

Hassan, G., Kirmayer, L., Mekki-Berrada, A., Quosh, C., El Chammay, R., Deville-Stoetzel, J. B., Youssef, A., Jefee-Bahloul, H., Barkeel-Oteo, A., Coutts, A., Song, S., & Ventevogel, P. (2015). *Culture, Context and the Mental Health and Psychosocial Wellbeing of Syrians: A Review for Mental Health and Psychosocial Support staff working with Syrians Affected by Armed Conflict.* UNHCR.

Heim, E., Mewes, R., Abi Ramia, J., Glaesmer, H., Hall, B., Harper Shehadeh, M., Ünlü, B., Kananian, S., Kohrt, B. A., Lechner-Meichsner, F., Lotzin, A., Moro, M. R., Radjack, R., Salamanca-Sanabria, A., Singla, D. R., Starck, A., Sturm, G., Tol, W., Weise, C., & Knaevelsrud, C. (2021). Reporting Cultural Adaptation in Psychological Trials – The RECAPT criteria. *Clinical Psychology in Europe*, *3*(Special Issue), e6351. https://doi.org/10.32872/cpe.6351

Hoell, A., Kourmpeli, E., Salize, H. J., Heinz, A., Padberg, F., Habel, U., Kamp-Becker, I., Höhne, E., Böge, K., & Bajbouj, M. (2021). Prevalence of depressive symptoms and symptoms of post-traumatic stress disorder among newly arrived refugees and asylum seekers in Germany: Systematic review and meta-analysis. *BJPsych Open*, *7*(3), e93. https://doi.org/10.1192/bjo.2021.54

Jannesari, S., Hatch, S., Prina, M., & Oram, S. (2020). Post-migration Social–Environmental Factors Associated with Mental Health Problems Among Asylum Seekers: A Systematic Review. *Journal of Immigrant and Minority Health*, *22*(5), 1055–1064. https://doi.org/10.1007/s10903-020-01025-2

Kaltenbach, E., Schauer, M., Hermenau, K., Elbert, T., & Schalinski, I. (2018). Course of Mental Health in Refugees—A One Year Panel Survey. *Frontiers in Psychiatry*, *9*, 352. https://doi.org/10.3389/fpsyt.2018.00352

Kiselev, N. (2020). Barriers to access to outpatient mental health care for refugees and asylum seekers in Switzerland: The therapist’s view. *BMC Psychiatry*, *20*(1), 20:378. https://doi.org/10.1186/s12888-020-02783-x

Kiselev, N., Pfaltz, M., Haas, F., Schick, M., Kappen, M., Sijbrandij, M., De Graaff, A. M., Bird, M., Hansen, P., Ventevogel, P., Fuhr, D. C., Schnyder, U., & Morina, N. (2020). Structural and socio-cultural barriers to accessing mental healthcare among Syrian refugees and asylum seekers in Switzerland. *European Journal of Psychotraumatology*, *11*(1), 1717825. https://doi.org/10.1080/20008198.2020.1717825

Li, M., & Anderson, J. G. (2016). Pre-migration Trauma Exposure and Psychological Distress for Asian American Immigrants: Linking the Pre- and Post-migration Contexts. *Journal of Immigrant and Minority Health*, *18*(4), 728–739. https://doi.org/10.1007/s10903-015-0257-2

Mediavilla, R., McGreevy, K. R., Felez-Nobrega, M., Monistrol-Mula, A., Bravo-Ortiz, M.-F., Bayón, C., Rodríguez-Vega, B., Nicaise, P., Delaire, A., Sijbrandij, M., Witteveen, A. B., Purgato, M., Barbui, C., Tedeschi, F., Melchior, M., Van Der Waerden, J., McDaid, D., Park, A.-L., Kalisch, R., … on behalf of the RESPOND Consortium. (2022). Effectiveness of a stepped-care programme of internet-based psychological interventions for healthcare workers with psychological distress: Study protocol for the RESPOND healthcare workers randomised controlled trial. *DIGITAL HEALTH*, *8*, 205520762211290. https://doi.org/10.1177/20552076221129084

Mesa-Vieira, C., Haas, A. D., Buitrago-Garcia, D., Roa-Diaz, Z. M., Minder, B., Gamba, M., Salvador, D., Gomez, D., Lewis, M., Gonzalez-Jaramillo, W. C., Pahud de Mortanges, A., Buttia, C., Muka, T., Trujillo, N., & Franco, O. H. (2022). Mental health of migrants with pre-migration exposure to armed conflict: A systematic review and meta-analysis. *The Lancet Public Health*, *7*(5), e469–e481. https://doi.org/10.1016/S2468-2667(22)00061-5

Miller, A., Hess, J. M., Bybee, D., & Goodkind, J. R. (2018). Understanding the mental health consequences of family separation for refugees: Implications for policy and practice. *American Journal of Orthopsychiatry*, *88*(1), 26–37. https://doi.org/10.1037/ort0000272

Miller, K. E., & Rasmussen, A. (2010). War exposure, daily stressors, and mental health in conflict and post-conflict settings: Bridging the divide between trauma-focused and psychosocial frameworks. *Social Science & Medicine*, *70*(1), 7–16. https://doi.org/10.1016/j.socscimed.2009.09.029

Miller, K. E., & Rasmussen, A. (2017). The mental health of civilians displaced by armed conflict: An ecological model of refugee distress. *Epidemiology and Psychiatric Sciences*, *26*(2), 129–138. https://doi.org/10.1017/S2045796016000172

Nezu, A. M., Nezu, C. M., & D’Zurilla, T. J. (2012). *Problem-solving therapy: A treatment manual. Springer publishing company.*

Nickerson, A., Steel, Z., Bryant, R., Brooks, R., & Silove, D. (2011). Change in visa status amongst Mandaean refugees: Relationship to psychological symptoms and living difficulties. *Psychiatry Research*, *187*(1–2), 267–274. https://doi.org/10.1016/j.psychres.2010.12.015

Ohaeri, J. U., & Awadalla, A. W. (2009). The reliability and validity of the short version of the WHO Quality of Life Instrument in an Arab general population. *Annals of Saudi Medicine*, *29*(2), 98–104. https://doi.org/10.4103/0256-4947.51790

Patanè, M., Ghane, S., Karyotaki, E., Cuijpers, P., Schoonmade, L., Tarsitani, L., & Sijbrandij, M. (2022). Prevalence of mental disorders in refugees and asylum seekers: A systematic review and meta-analysis. *Global Mental Health*, *9*, 250–263. https://doi.org/10.1017/gmh.2022.29

Porter, M., & Haslam, N. (2005). Predisplacement and Postdisplacement Factors Associated With Mental Health of Refugees and Internally Displaced Persons: A Meta-analysis. *JAMA*, *294*(5), 602. https://doi.org/10.1001/jama.294.5.602

Purgato, M., Gastaldon, C., Papola, D., van Ommeren, M., Barbui, C., & Tol, W. A. (2018). Psychological therapies for the treatment of mental disorders in low- and middle-income countries affected by humanitarian crises. *Cochrane Database of Systematic Reviews*, *2018*(7). https://doi.org/10.1002/14651858.CD011849.pub2

Renner, A., Hoffmann, R., Nagl, M., Roehr, S., Jung, F., Grochtdreis, T., König, H.-H., Riedel-Heller, S., & Kersting, A. (2020). Syrian refugees in Germany: Perspectives on mental health and coping strategies. *Journal of Psychosomatic Research*, *129*, 109906. https://doi.org/10.1016/j.jpsychores.2019.109906

Rohlof, H. G., Knipscheer, J. W., & Kleber, R. J. (2014). Somatization in refugees: A review. *Social Psychiatry and Psychiatric Epidemiology*, *49*(11), 1793–1804. https://doi.org/10.1007/s00127-014-0877-1

Schick, M., Morina, N., Mistridis, P., Schnyder, U., Bryant, R. A., & Nickerson, A. (2018). Changes in Post-migration Living Difficulties Predict Treatment Outcome in Traumatized Refugees. *Frontiers in Psychiatry*, *9*, 476. https://doi.org/10.3389/fpsyt.2018.00476

Schick, M., Zumwald, A., Knöpfli, B., Nickerson, A., Bryant, R. A., Schnyder, U., Müller, J., & Morina, N. (2016). Challenging future, challenging past: The relationship of social integration and psychological impairment in traumatized refugees. *European Journal of Psychotraumatology*, *7*(1), 28057. https://doi.org/10.3402/ejpt.v7.28057

Schulz, A., Stolz, T., Vincent, A., Krieger, T., Andersson, G., & Berger, T. (2016). A sorrow shared is a sorrow halved? A three-arm randomized controlled trial comparing internet-based clinician-guided individual versus group treatment for social anxiety disorder. *Behaviour Research and Therapy*, *84*, 14–26. https://doi.org/10.1016/j.brat.2016.07.001

Seewer, N., Skoko, A., Käll, A., Andersson, G., Luhmann, M., Berger, T., & Krieger, T. (2024). Efficacy of an Internet-based self-help intervention with human guidance or automated messages to alleviate loneliness: A three-armed randomized controlled trial. *Scientific Reports*, *14*(1), 6569. https://doi.org/10.1038/s41598-024-57254-0

Sengoelge, M., Nissen, A., & Solberg, Ø. (2022). Post-Migration Stressors and Health-Related Quality of Life in Refugees from Syria Resettled in Sweden. *International Journal of Environmental Research and Public Health*, *19*(5), 2509. https://doi.org/10.3390/ijerph19052509

Shannon, P. J., Wieling, E., Simmelink-McCleary, J., & Becher, E. (2015). Beyond Stigma: Barriers to Discussing Mental Health in Refugee Populations. *Journal of Loss and Trauma*, *20*(3). https://doi.org/10.1080/15325024.2014.934629

Sijbrandij, M., Acarturk, C., Bird, M., Bryant, R. A., Burchert, S., Carswell, K., de Jong, J., Dinesen, C., Dawson, K. S., El Chammay, R., van Ittersum, L., Jordans, M., Knaevelsrud, C., McDaid, D., Miller, K., Morina, N., Park, A.-L., Roberts, B., van Son, Y., … Cuijpers, P. (2017). Strengthening mental health care systems for Syrian refugees in Europe and the Middle East: Integrating scalable psychological interventions in eight countries. *European Journal of Psychotraumatology*, *8*(sup2), 1388102. https://doi.org/10.1080/20008198.2017.1388102

Silove, D. (2013). The ADAPT model: A conceptual framework for mental health and psychosocial programming in post conflict settings. *Intervention*, *11*(3), 237–248. https://doi.org/10.1097/WTF.0000000000000005

Silove, D., Sinnerbrink, I., Field, A., Manicavasagar, V., & Steel, Z. (1997). Anxiety, depression and PTSD in asylum-seekers: Assocations with pre-migration trauma and post-migration stressors. *British Journal of Psychiatry*, *170*(4), 351–357. https://doi.org/10.1192/bjp.170.4.351

Silove, D., Ventevogel, P., & Rees, S. (2017). The contemporary refugee crisis: An overview of mental health challenges. *World Psychiatry*, *16*(2), 130–139. https://doi.org/10.1002/wps.20438

Skevington, S. M., Lotfy, M., & O’Connell, K. A. (2004). The World Health Organization’s WHOQOL-BREF quality of life assessment: Psychometric properties and results of the international field trial. A Report from the WHOQOL Group. *Quality of Life Research*, *13*(2), 299–310. https://doi.org/10.1023/B:QURE.0000018486.91360.00

Spaaij, J., Kiselev, N., Berger, C., Bryant, R. A., Cuijpers, P., de Graaff, A. M., Fuhr, D. C., Hemmo, M., McDaid, D., Moergeli, H., Park, A.-L., Pfaltz, M. C., Schick, M., Schnyder, U., Wenger, A., Sijbrandij, M., & Morina, N. (2022). Feasibility and acceptability of Problem Management Plus (PM+) among Syrian refugees and asylum seekers in Switzerland: A mixed-method pilot randomized controlled trial. *European Journal of Psychotraumatology*, *13*(1), 2002027. https://doi.org/10.1080/20008198.2021.2002027

Staatssekretariat für Migration, S. (2023). *Kommentierte Asylstatistik 2022*. https://www.google.com/url?sa=t&rct=j&q=&esrc=s&source=web&cd=&ved=2ahUKEwjxx87l2p6CAxWkgf0HHU8SClUQFnoECAgQAw&url=https%3A%2F%2Fwww.sem.admin.ch%2Fdam%2Fsem%2Fde%2Fdata%2Fpubliservice%2Fstatistik%2Fasylstatistik%2F2022%2Fstat-jahr-2022-kommentar.pdf&usg=AOvVaw3D5MVJLhzRUMq5zeyCM-Y0&opi=89978449

Staatssekretariat für Migration, S. (2025). *Kommentierte Asylstatistik 2024*. https://www.sem.admin.ch/dam/sem/de/data/publiservice/statistik/asylstatistik/2024/stat-jahr-2024-kommentar.pdf

Steel, Z., Chey, T., Silove, D., Marnane, C., Bryant, R. A., & van Ommeren, M. (2009). Association of Torture and Other Potentially Traumatic Events With Mental Health Outcomes Among Populations Exposed to Mass Conflict and Displacement: A Systematic Review and Meta-analysis. *JAMA*, *302*(5), 537. https://doi.org/10.1001/jama.2009.1132

Thompson, C. T., Vidgen, A., & Roberts, N. P. (2018). Psychological interventions for post-traumatic stress disorder in refugees and asylum seekers: A systematic review and meta-analysis. *Clinical Psychology Review*, *63*, 66–79. https://doi.org/10.1016/j.cpr.2018.06.006

Tol, W. A., Leku, M. R., Lakin, D. P., Carswell, K., Augustinavicius, J., Adaku, A., Au, T. M., Brown, F. L., Bryant, R. A., Garcia-Moreno, C., Musci, R. J., Ventevogel, P., White, R. G., & van Ommeren, M. (2020). Guided self-help to reduce psychological distress in South Sudanese female refugees in Uganda: A cluster randomised trial. *The Lancet Global Health*, *8*(2), e254–e263. https://doi.org/10.1016/S2214-109X(19)30504-2

Turrini, G., Purgato, M., Acarturk, C., Anttila, M., Au, T., Ballette, F., Bird, M., Carswell, K., Churchill, R., Cuijpers, P., Hall, J., Hansen, L. J., Kösters, M., Lantta, T., Nosè, M., Ostuzzi, G., Sijbrandij, M., Tedeschi, F., Valimaki, M., … Barbui, C. (2019). Efficacy and acceptability of psychosocial interventions in asylum seekers and refugees: Systematic review and meta-analysis. *Epidemiology and Psychiatric Sciences*, *28*(04), 376–388. https://doi.org/10.1017/S2045796019000027

Turrini, G., Purgato, M., Ballette, F., Nosè, M., Ostuzzi, G., & Barbui, C. (2017). Common mental disorders in asylum seekers and refugees: Umbrella review of prevalence and intervention studies. *International Journal of Mental Health Systems*, *11*(1), 51. https://doi.org/10.1186/s13033-017-0156-0

van der Boor, C. F., Amos, R., Nevitt, S., Dowrick, C., & White, R. G. (2020). Systematic review of factors associated with quality of life of asylum seekers and refugees in high-income countries. *Conflict and Health*, *14*(1), 14:48. https://doi.org/10.1186/s13031-020-00292-y

WHOQOL-Group. (1998). Development of the World Health Organization WHOQOL-BREF quality of life assessment. *Psychological Medicine*, *28*(3), 551–558. https://doi.org/10.1017/S0033291798006667

World Health Organization. (2020). *Doing what matters in times of stress: An illustrated guide*. World Health Organization. https://apps.who.int/iris/handle/10665/331901
